# Off-target structural insights: ArnA and AcrB in bacterial membrane-protein cryo-EM analysis

**DOI:** 10.1107/S2059798325007089

**Published:** 2025-09-10

**Authors:** Mehmet Caliseki, Ufuk Borucu, Sathish K. N. Yadav, Christiane Schaffitzel, Burak Veli Kabasakal

**Affiliations:** a Turkish Accelerator and Radiation Laboratory, 06830Ankara, Türkiye; b Department of Molecular Biology, Genetics and Bioengineering, Graduate School of Engineering and Natural Sciences Sabancı University 34420Istanbul Türkiye; c School of Biochemistry University of Bristol 1 Tankard’s Close BristolBS8 1TD United Kingdom; d Department of Biological Sciences Middle East Technical University 06800Ankara Türkiye; University of Queensland, Australia

**Keywords:** cryo-EM, ArnA, AcrB, membrane-protein expression, protein quality control

## Abstract

Membrane-protein quality control in *Escherichia coli* involves coordinated actions of the AAA+ protease FtsH, the insertase YidC and the regulatory complex HflKC. These systems maintain proteostasis by facilitating membrane-protein insertion, folding and degradation. To gain structural insights into a putative complex formed by FtsH and YidC, we performed single-particle cryogenic electron microscopy on detergent-solubilized membrane samples, from which FtsH and YidC were purified using Ni–NTA affinity and size-exclusion chromatography. Although SDS–PAGE analysis indicated high purity of these proteins, cryo-EM data sets unexpectedly yielded high-resolution structures of ArnA and AcrB at 4.0 and 2.9 Å resolution, respectively. ArnA is a bifunctional enzyme involved in lipid A modification and polymyxin resistance, while AcrB is a multidrug efflux transporter of the AcrAB–TolC system. ArnA and AcrB, known Ni–NTA purification contaminants, were also consistently detected by mass spectrometry in Strep-Tactin affinity-purified samples, validating their presence independently of affinity-tag selection. ArnA, which is typically cytoplasmic, was consistently found in membrane-isolated samples, indicating an association with membrane components. Only 2D class averages corresponding to the cytoplasmic AAA+ domain of FtsH were observed; neither side views of full-length FtsH nor densities corresponding to an intact FtsH–YidC complex could be identified, due to the conformational flexibility of the FtsH complex and its transient interaction with YidC, which limited particle alignment and stable classification in cryo-EM data sets. Two-dimensional class averages revealed additional particles resembling GroEL and cytochrome *bo*_3_ oxidase. These results underscore the utility of cryo-EM in uncovering off-target yet structurally well defined complexes, which may reflect physiologically relevant interactions or purification biases during membrane-protein overexpression.

## Abstract 2

This study highlights that cryo-EM can reveal structural information beyond the intended target, and that overexpression of membrane proteins involved in quality control may lead to the co-purification of proteins such as ArnA and AcrB.

## 1. Introduction

Bacterial membrane proteostasis relies on the coordinated action of membrane-protein biogenesis and degradation pathways, which ensure the proper insertion, folding and removal of membrane proteins (Akiyama, 2009 ▸; Watkins *et al.*, 2022 ▸; Njenga *et al.*, 2023 ▸). Membrane-protein quality control plays a central role in this system by preventing the accumulation of misfolded or nonfunctional proteins that could compromise membrane integrity and disrupt cellular function (Akiyama, 2009 ▸; Dalbey *et al.*, 2012 ▸; Liu *et al.*, 2020 ▸; Njenga *et al.*, 2023 ▸; Watkins *et al.*, 2022 ▸; Ero *et al.*, 2024 ▸).

In Gram-negative bacteria, inner membrane-protein quality control involves distinct systems responsible for protein insertion and degradation. The membrane-bound AAA+ protease FtsH removes misfolded or unassembled membrane proteins in an ATP- and Zn^2+^-dependent manner (Akiyama, 2002 ▸; Ito & Akiyama, 2005 ▸; Bieniossek *et al.*, 2006 ▸; Langklotz *et al.*, 2012 ▸; Carvalho *et al.*, 2021 ▸). FtsH forms a complex with the membrane proteins HflKC, which modulate its proteolytic activity and substrate specificity (Kihara *et al.*, 1998 ▸; Saikawa *et al.*, 2004 ▸; Qiao *et al.*, 2022 ▸; Ma *et al.*, 2022 ▸; Akkulak *et al.*, 2024 ▸; Ghanbarpour *et al.*, 2025 ▸). In parallel, the insertase YidC facilitates the membrane integration and proper folding of nascent polypeptides, functioning either independently or in association with the Sec translocon (Houben *et al.*, 2000 ▸; van der Laan *et al.*, 2005 ▸; Beck *et al.*, 2001 ▸; Schulze *et al.*, 2014 ▸; Dalbey *et al.*, 2014 ▸; Komar *et al.*, 2016 ▸; Botte *et al.*, 2016 ▸; Petriman *et al.*, 2018 ▸; Shanmugam *et al.*, 2019 ▸; Caliseki *et al.*, 2025 ▸). These systems act together to maintain membrane proteostasis and prevent the accumulation of defective protein species under both normal and stress conditions (van Bloois *et al.*, 2008 ▸; Akiyama, 2009 ▸; Njenga *et al.*, 2023 ▸; Caliseki, Schaffitzel *et al.*, 2025 ▸; Caliseki, Zorman *et al.*, 2025 ▸).

Although previous studies have provided biochemical evidence for an interaction between FtsH and YidC (Caliseki, Zorman *et al.*, 2025 ▸; van Bloois *et al.*, 2008 ▸), their potential structural association has remained unresolved. To gain structural insight into their interplay, we carried out single-particle cryogenic electron microscopy (cryo-EM) on detergent-solubilized membrane fractions purified via affinity and size-exclusion chromatography.

Unexpectedly, the data set yielded high-resolution reconstructions of two unrelated proteins: ArnA and AcrB. ArnA is a bifunctional enzyme that catalyzes the biosynthesis of 4-amino-4-deoxy-l-arabinose (l-Ara4N), a nucleotide-activated sugar that is incorporated into lipid A to reduce its net negative charge and thereby enhance resistance to cationic antimicrobial peptides such as polymyxins (Gatzeva-Topalova *et al.*, 2005 ▸; Williams *et al.*, 2005 ▸; Yang *et al.*, 2019 ▸). AcrB is a key component of the AcrAB–TolC multidrug efflux system, responsible for exporting diverse substrates across the bacterial envelope (Murakami *et al.*, 2002 ▸; Seeger *et al.*, 2006 ▸; Takatsuka *et al.*, 2010 ▸; Trinh *et al.*, 2023 ▸). ArnA and AcrB are known contaminants in Ni–NTA affinity purification (Bolanos-Garcia & Davies, 2006 ▸; Veesler *et al.*, 2008 ▸; Glover *et al.*, 2011 ▸; Andersen *et al.*, 2013 ▸). Nevertheless, in a parallel study, we also detected these proteins in Strep-Tactin purified fractions by mass spectrometry, suggesting that their presence may reflect a specific association rather than nonspecific contamination (Caliseki, Zorman *et al.*, 2025 ▸). Additionally, two-dimensional (2D) class averages in the cryo-EM data set revealed distinct particles resembling GroEL (Fujita *et al.*, 2023 ▸) and cytochrome *bo*_3_ oxidase (Su *et al.*, 2021 ▸).

This unexpected outcome highlights the ability of cryo-EM to uncover off-target yet structurally well defined complexes, providing insight into biologically relevant proteins beyond the intended target, and underlining both the sensitivity and the interpretative challenges of the technique.

## 2. Materials and methods

### 2.1. Expression systems and plasmid design

The FYHC Co-T expression system was used to co-express FtsH, YidC, HflK and HflC in *Escherichia coli* C43 (DE3) cells (Caliseki *et al.*, 2025 ▸). This system employed the pETDuet-1 (Novagen, catalogue No. 71146-3) and pRSFDuet-1 (Novagen, catalogue No. 71341) plasmids, both driven by T7 promoters. The pETDuet-1 vector encoded YidC with a C-terminal 10×His tag and FtsH with a C-terminal 3×StrepTag II, while the pRSFDuet-1 plasmid carried untagged HflK and HflC. Ampicillin and kanamycin resistance markers enabled co-transformation and stable maintenance of both plasmids for simultaneous expression of the target proteins.

### 2.2. Membrane-protein expression, isolation and purification

*E. coli* C43 (DE3) cells harboring the FYHC Co-T expression system were used for recombinant protein expression. Starter cultures were grown overnight at 37°C in Luria–Bertani (LB) medium containing appropriate antibiotics. A 5 ml aliquot was transferred into 2 l baffled flasks containing 500 ml Terrific Broth (TB; Sigma–Aldrich, catalogue No. T0918). Typical culture volumes were 6 l. Cultures were incubated at 37°C until an optical density at 600 nm (OD_600_) of ∼0.8 was reached. Protein expression was induced by the addition of 0.4 m*M* isopropyl β-d-1-thiogalactopyranoside (IPTG) and the cultures were incubated at 18°C for 18 h.

The cells were harvested by centrifugation at 5000*g* for 30 min at 4°C and resuspended in lysis buffer (20 m*M* HEPES pH 7.5, 150 m*M* NaCl, 8% glycerol, 2 m*M* MgCl_2_) supplemented with 1 m*M* phenylmethylsulfonyl fluoride (PMSF; Roche, catalogue No. 11359061001), one tablet of EDTA-free protease-inhibitor cocktail (Pierce, catalogue No. A32965) and 1 µg ml^−1^ Benzonase nuclease (Millipore, catalogue No. E1014). The cell pellet was resuspended and incubated for 1 h at 4°C with gentle agitation to allow complete resuspension and enzyme activity. The cells were then lysed by sonication on ice using a probe sonicator at 40% amplitude, with 2 s on/8 s off cycles for 2 min, repeated twice. The lysate was clarified by centrifugation at 10 000*g* for 1.5 h at 4°C.

Membrane fractions were isolated from the lysate by ultra­centrifugation at 100 000*g* for 1 h at 4°C. The resulting pellets were resuspended in a solubilization buffer composed of 20 m*M* HEPES pH 7.5, 150 m*M* NaCl, 8%(*v*/*v*) glycerol, 2 m*M* MgCl_2_ supplemented with detergents. For the first and second cryo-EM screenings, 1%(*w*/*v*) *n*-dodecyl-β-d-maltoside (DDM; Anatrace, catalogue No. D310A) was added to the solubilization buffer. For the third screening, the buffer was further supplemented with 0.1%(*w*/*v*) cholesterol hemisuccinate (CHS; Anatrace, catalogue No. CH210) in addition to 1% DDM to reduce aggregation and enhance membrane-protein stability. Solubilization was performed at 4°C for 1 h with gentle agitation.

Subsequently, solubilized membrane proteins were chemically cross-linked by adding 0.25 m*M* dithiobis(succinimidyl propionate) (DSP) for the first cryo-EM screening or 1 m*M* DSP for the second and third screenings, followed by incubation at 4°C for 2 h. The reaction was quenched by the addition of 20 m*M* Tris–HCl pH 7.5 and incubated on ice for 15 min. Insoluble material was removed by ultracentrifugation at 100 000*g* for 1 h at 4°C. The resulting supernatant was clarified by filtration through a 0.45 µm membrane prior to affinity purification.

Solubilized and cross-linked membrane proteins were first subjected to Ni–NTA affinity purification using the gravity-flow technique. For the first and second cryo-EM screenings, the purification buffers contained 0.02%(*w*/*v*) DDM. For the third screening, the buffer composition was modified to include 0.05%(*w*/*v*) DDM and 0.005%(*w*/*v*) CHS to improve protein stability. Size-exclusion chromatography (SEC) was subsequently performed on an ÄKTA micro FPLC system (Cytiva) using a Superose 6 Increase 10/300 GL column equilibrated in the corresponding detergent-containing buffer.

Ni–NTA resin (HisPur Superflow Agarose, Thermo Scientific, catalogue No. 25215) was equilibrated with binding buffer composed of 20 m*M* HEPES pH 7.5, 150 m*M* NaCl, 8%(*v*/*v*) glycerol, 2 m*M* MgCl_2_, the appropriate concentration of DDM and 10 m*M* imidazole. After protein binding, the resin was washed sequentially with high-salt buffer (including 1 *M* NaCl) and wash buffer containing 50 m*M* imidazole. Bound proteins were eluted using elution buffer containing 300 m*M* imidazole.

Eluted fractions were collected and diluted 3:1 with imidazole-free buffer (20 m*M* HEPES pH 7.5, 150 m*M* NaCl, 8% glycerol, 2 m*M* MgCl_2_, appropriate DDM concentration) to reduce the imidazole concentration prior to concentration. The diluted samples were then concentrated using Amicon Ultra-15 Centrifugal Filter Units (100 kDa MWCO; Merck Millipore, catalogue No. UFC910024) at 4°C by centrifugation at 4000*g* using a swinging-bucket rotor. Concentration was continued until the final volume reached approximately 1.5 ml with a protein concentration of ∼6–8 mg ml^−1^.

Concentrated samples were subjected to SEC using an ÄKTA micro system (Cytiva) equipped with a Superose 6 Increase 3.2/300 column (Cytiva, catalogue No. 29091598) pre-equilibrated with buffer consisting of 20 m*M* HEPES pH 7.5, 100 m*M* NaCl, 2 m*M* MgCl_2_. For the first and second cryo-EM screenings, the SEC buffer contained 0.02%(*w*/*v*) DDM. For the third sample, the buffer was supplemented with 0.05%(*w*/*v*) DDM and 0.005%(*w*/*v*) CHS. A total of 50 µl sample was injected per run, and separation was carried out at a flow rate of 0.05 ml min^−1^.

### 2.3. SDS–PAGE and Western blot analysis

Protein expression and purification profiles were assessed by SDS–PAGE (Supplementary Figs. S1–S3) and Western blotting (Supplementary Fig. S1). Electrophoresis was performed using commercial 4–12% Bolt Bis-Tris Plus Mini Gels (Thermo Fisher Scientific, catalogue No. NW04125BOX) at 180 V in Tris–glycine running buffer (25 m*M* Tris, 192 m*M* glycine, 0.1% SDS pH 8.3). After separation, proteins were visualized by Coomassie Brilliant Blue staining.

For Western blotting, proteins were transferred from the SDS gels onto 0.2 µm nitrocellulose membranes (Bio-Rad) using the Trans-Blot Turbo system (Bio-Rad) at 25 V for 7 min. Membranes were blocked with 3%(*w*/*v*) BSA in TBS-T [20 m*M* Tris–HCl pH 7.5, 150 m*M* NaCl, 0.1%(*v*/*v*) Tween-20] for 1 h at room temperature. Tagged proteins were detected using HRP-conjugated primary antibodies: Anti-His (Qiagen, 1:5000), anti-StrepTag II (Sigma–Aldrich, 1:5000) and anti-Flag (Rockland, 1:10 000). Following antibody incubation and washing, chemiluminescence signals were developed with Pierce ECL substrate (Thermo Scientific; Supplementary Fig. S1).

### 2.4. Negative-stain (NS) electron microscopy

SEC-purified samples were diluted into SEC buffer to final protein concentrations of approximately 0.05, 0.025 or 0.01 mg ml^−1^. A 4 µl aliquot was applied onto glow-discharged carbon-coated copper grids (CF300-CU, 300 mesh; Electron Microscopy Sciences) and incubated for 1 min at room temperature. Excess sample was blotted off with filter paper.

Grids were stained with 2%(*w*/*v*) uranyl acetate for 1 min, followed by blotting with filter paper. A second drop of uranyl acetate was applied to wash the grid and excess stain was again removed with filter paper. Grids were left to air-dry completely before imaging.

NS-EM images were acquired using a Tecnai T12 transmission electron microscope (Thermo Fisher Scientific) operated at 120 kV equipped with a Ceta 16 M CCD detector at the Wolfson Bioimaging Facility, University of Bristol at magnifications ranging from 25 000× to 86 000×.

### 2.5. Cryo-EM grid preparation, screening and data collection

Cryo-EM grids were prepared using SEC-purified samples obtained under three different preparation conditions (see above). In the first screening, the grids were vitrified using a Leica EM GP2 system, while in the second and third screenings the grids were vitrified using a Vitrobot Mark IV system (Thermo Fisher Scientific). Prior to sample application, the grids were rendered hydrophilic using an Elmo glow-discharge unit (Cordouan Technologies) at a setting of 300 mC. A 4 µl aliquot of each sample was applied to glow-discharged R2.2/2 nm carbon-coated 300 mesh copper grids (Quantifoil). The initial cryo-EM grid preparation involved grids vitrified using the Leica EM GP2 system and screened on a Talos Arctica, with samples cross-linked using 0.25 m*M* DSP cross-linker. The second and third cryo-EM screenings employed grids with 1 m*M* DSP cross-linked samples vitrified using the Vitrobot Mark IV system under the following conditions: 4°C, 100% relative humidity, blot force of 0 or 1, 10 s wait time, 2 s blot time, no drain time and back-side blotting enabled. For the first data set from the second screening, 0.4%(*w*/*v*) CHAPS was added prior to vitrification to reduce aggregation. In the second data set from the third screening, 0.05%(*w*/*v*) DDM was used with 0.005%(*w*/*v*) CHS maintained in the final SEC buffer.

Cryo-EM grid screening and data collection were conducted in the GW4 Facility for High-Resolution Electron Cryo-Microscopy at the University of Bristol using a Talos Arctica transmission electron microscope (Thermo Fisher Scientific) operating at 200 kV equipped with an energy filter and a K2 direct electron detector (Gatan). No data were collected from the first screening due to severe sample aggregation (Supplementary Fig. S4). The first data set (3908 movies) and second data set (7506 movies) were obtained from the second and third grid screenings, respectively. Both data sets were recorded at 130 000× nominal magnification with a calibrated pixel size of 1.05 Å, 6 s exposure per movie (60 frames) and total electron doses of 58.2 and 60.6 e^−^ Å^−2^, respectively (Table 1 ▸).

### 2.6. Cryo-EM data processing

**Figure 1:**
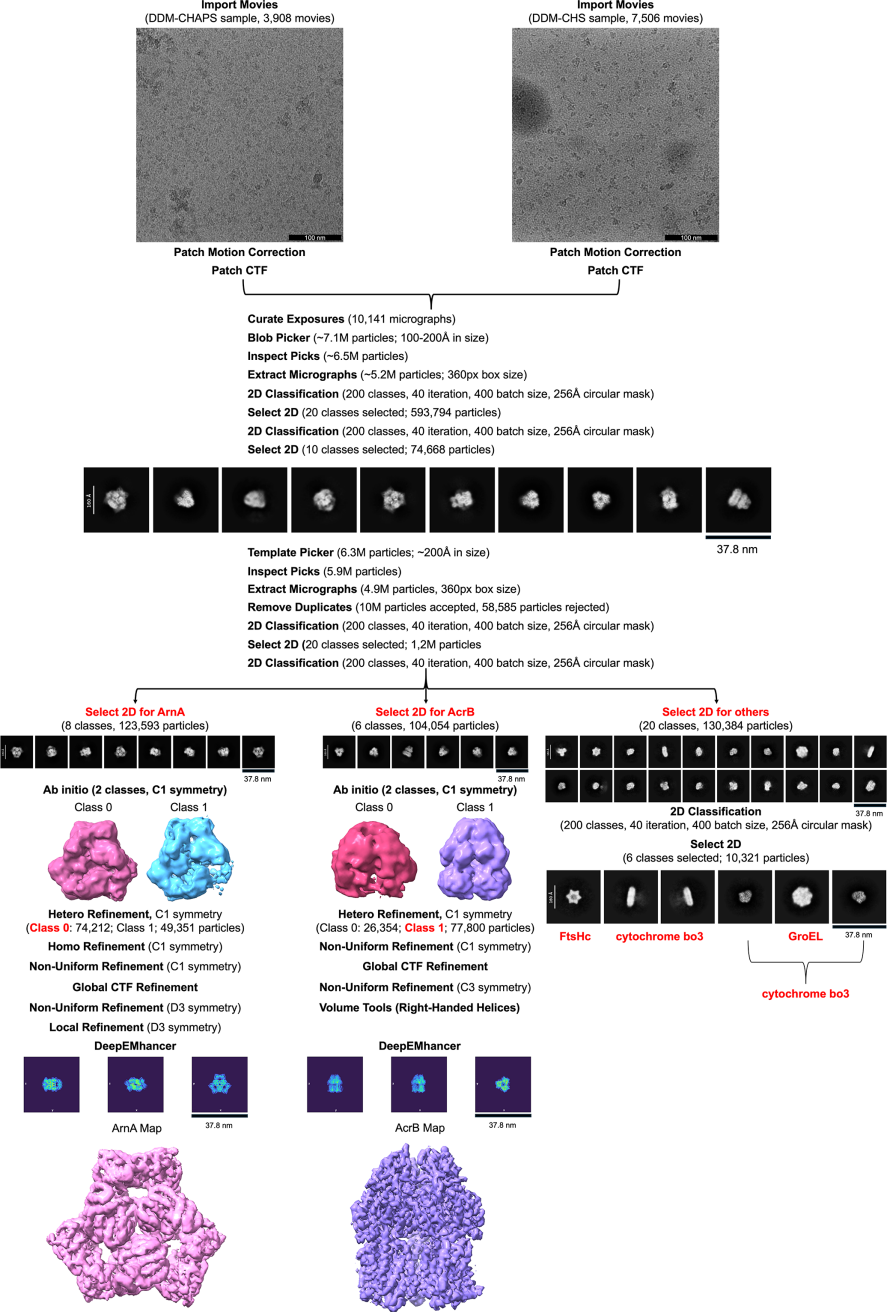
Optimized *cryoSPARC* workflow using the combined cryo-EM data set. Two cryo-EM data sets were collected from DSP-cross-linked membrane-protein samples. The first data set (3908 movies) was prepared with 0.02% DDM and 0.4%(*w*/*v*) CHAPS as an additive, and the second data set (7506 movies) with 0.05% DDM and 0.005%(*w*/*v*) cholesterol hemisuccinate (CHS). Motion correction and CTF estimation were performed separately for each data set. 10 141 micrographs were used for particle picking, resulting in 7.13 million particles (estimated diameter 100–200 Å). Two rounds of 2D classification (200 classes, 256 Å mask, 40 online EM iterations, batch size 400) retained 74 668 particles (scale bar 37.8 nm). These classes were used as templates for template-based picking, which yielded 6.3 million particles. After re-extraction and duplicate removal, a final set of 10.13 million unique particles was obtained. 2D classification yielded 1.26 million particles distributed across the best 20 classes. For ArnA, 123 593 particles corresponding to ArnA 2D classes (scale bar 37.8 nm for 2D class averages) were selected and processed through *ab initio* reconstruction with *C*1 symmetry, followed by heterogeneous and non-uniform refinement using *D*3 symmetry. For AcrB, 104 054 particles corresponding to AcrB 2D classes (scale bar 37.8 nm for 2D class averages) were selected and refined using *C*3 symmetry. Global CTF refinement and *DeepEMhancer* post-processing were applied to both maps. Handedness correction for AcrB was performed using the *Volume Tools* utility in *cryoSPARC*. Particles corresponding to GroEL, cytochrome *bo*_3_ oxidase and other minor components were also detected during classification (scale bar 37.8 nm for 2D class averages).

Cryo-EM image processing was performed using *cryo­SPARC* (Punjani *et al.*, 2017 ▸). Movies from both data sets were initially processed independently (Fig. 1 ▸). Patch motion correction and patch CTF estimation (Downing & Glaeser, 2008 ▸; Punjani *et al.*, 2017 ▸) were performed separately, followed by micrograph curation.

For the first data set, particle picking in *cryoSPARC* (Punjani *et al.*, 2017 ▸) yielded 671 626 particles, of which 44 881 were retained after 2D classification (Supplementary Fig. S4*a*). For the second data set, 2 123 935 particles were extracted using the same 360-pixel box size. After 2D classification, 185 213 particles were retained (Supplementary Fig. S4*b*).

As similar 2D class averages were observed in both data sets, the micrographs were merged at the CTF refinement stage for joint downstream processing. From the combined data set, 10 141 curated micrographs were selected. Initial particle picking was performed using blob-based algorithms with estimated particle diameters between 100 and 200 Å, yielding approximately 7.13 million particles. After particle picking, low-quality particles were removed based on 2D class averages, yielding a curated data set of 6.57 million particles.

Subsequently, two rounds of 2D classification were then performed using a circular mask diameter of 256 Å. The first round yielded 593 794 particles across 20 selected classes. The second round refined this to 74 668 particles from ten well defined classes, which were then used for template-based particle picking. This step identified 6.3 million particles. After combining with the initial blob-picked set and removing duplicates, 10.13 million unique particles were retained. A final round of 2D classification resulted in a cleaned data set containing 1.26 million particles for downstream processing (Fig. 1 ▸). This reduction from millions of initially picked particles to a smaller subset was driven by stringent quality filtering. Most of the excluded 2D classes displayed poor resolution, lacked clear structural features or showed severe anisotropy, and were therefore omitted from further analysis (Supplementary Fig. S7).

2D class averages revealed the presence of multiple co-purified proteins, including ArnA, AcrB, GroEL, cytochrome *bo*_3_ oxidase and the cytoplasmic domain of FtsH. Among the total data set, 130 384 particles were assigned to classes not matching ArnA or AcrB. These particles were further analyzed by 2D classification to group them based on structural similarity, using the same parameters described previously.

For ArnA, a total of 123 593 particles were selected and processed using *ab initio* reconstruction followed by heterogeneous, homogeneous and non-uniform refinement in *cryoSPARC* with *C*1 symmetry. In the final stage, *D*3 symmetry was imposed during the second non-uniform refinement (Punjani *et al.*, 2020 ▸). The resulting map was sharpened using *DeepEMhancer* (Sanchez-Garcia *et al.*, 2021 ▸).

For AcrB, 104 054 particles were subjected to the same processing workflow. Initial *ab initio* reconstruction and subsequent heterogeneous and non-uniform refinement were performed with *C*1 symmetry. *C*3 symmetry was applied during the final non-uniform refinement step (Punjani *et al.*, 2020 ▸). Handedness correction was conducted using volume tools within *cryoSPARC*. The final map was sharpened using *DeepEMhancer* (Sanchez-Garcia *et al.*, 2021 ▸).

The Fourier shell correlation (FSC) curves for ArnA and AcrB were calculated using *phenix.mtriage* (Afonine *et al.*, 2018 ▸; Liebschner *et al.*, 2019 ▸) based on the final map and the half-maps generated in *cryoSPARC*.

### 2.7. Model building and validation

Atomic model building and validation of the ArnA and AcrB cryo-EM maps were performed using an integrated modeling workflow. The initial fitting of atomic models into the experimental maps was carried out using *MOLREP* (Vagin & Teplyakov, 2010 ▸) within the *CCP*4 suite (Agirre *et al.*, 2023 ▸). The template structures PDB entry 6pih for ArnA (Yang *et al.*, 2019 ▸) and PDB entry 7rr7 for AcrB (Trinh *et al.*, 2023 ▸) were used for molecular replacement.

Subsequent real-space refinement was performed using *Phenix* (Liebschner *et al.*, 2019 ▸), incorporating local grid sampling, map-weighted geometry restraints and secondary-structure preservation. To improve global stereochemistry and atomic displacement parameters, an additional round of refinement was conducted using *REFMAC* as implemented in *CCP-EM* (Burnley *et al.*, 2017 ▸). Manual model adjustment, including side-chain placement, rotamer correction and loop rebuilding, was carried out in *Coot* (Emsley *et al.*, 2010 ▸).

All final models were validated using the comprehensive validation tools in *Phenix*, including *MolProbity* scores, Ramachandran outlier detection and map-to-model correlation metrics (Liebschner *et al.*, 2019 ▸; Table 2 ▸).

## 3. Results

### 3.1. Initial cryo-EM screening reveals aggregation in FtsH–YidC samples without additives

The first cryo-EM screening was carried out to investigate whether the sample was suitable for structural analysis of a potential FtsH–YidC complex. Membranes were solubilized in 1% *n*-dodecyl-β-d-maltoside (DDM) and cross-linked with 0.25 m*M* dithiobis(succinimidyl propionate) (DSP), followed by Ni–NTA affinity purification and size-exclusion chromatography (SEC) without the inclusion of stabilizing additives. Western blot analysis confirmed the presence of His-tagged YidC and Strep-tagged FtsH in SEC fractions (Supplementary Figs. S1*a*–S1*c*). The major SEC peak eluting at approximately 1.6 ml was collected for further analysis, and SDS–PAGE confirmed enrichment of the target proteins in this fraction (Supplementary Figs. S1*d* and S1*e*). Negative-stain electron microscopy revealed a homogeneous distribution of particles without obvious aggregation (Supplementary Fig. S1*f*). In contrast, cryo-EM grids vitrified using a Leica EM GP2 system and screened on a Talos Arctica microscope exhibited extensive particle aggregation within the vitreous ice, preventing further structural analysis (Supplementary Fig. S4). The observed clustering in cryo-EM micrographs could be attributed to the vitrification conditions, prompting further optimization of this step in subsequent preparations.

### 3.2. CHAPS improves particle dispersion in cross-linked FtsH–YidC samples

A second cryo-EM screening was performed after SEC purification of the DSP-cross-linked FtsH–YidC sample (Supplementary Figs. S2 and S4*a*). In this preparation, 0.4%(*w*/*v*) CHAPS was included to enhance particle dispersion and reduce aggregation. As a zwitterionic detergent, CHAPS was used to stabilize membrane-protein assemblies by minimizing the hydrophobic interactions that typically lead to aggregation during grid preparation (Kampjut *et al.*, 2021 ▸). Compared with the first screening, this approach resulted in improved vitrification quality, as shown by reduced aggregation and a more homogeneous particle distribution (Fig. 1 ▸ and Supplementary Fig. S4*a*). A total of 3908 micrographs were collected for this data set. Despite the improved sample quality, the number of usable particles and their angular distribution remained insufficient for high-resolution reconstruction (Supplementary Fig. S4*b*), indicating the need for further cryo-EM data collection.

### 3.3. Enhanced cryo-EM grid quality achieved through DDM–CHS solubilization

A final cryo-EM screening was performed using the C6 fraction obtained after SEC purification of the DSP-cross-linked FtsH–YidC sample (Supplementary Figs. S3*b* and S4*c*). In this experiment, membrane proteins were solubilized using a buffer containing 1%(*w*/*v*) DDM and 0.1%(*w*/*v*) CHS. CHS was included to enhance the structural stability of detergent-solubilized membrane proteins and reduce aggregation during vitrification, particularly considering that FtsH homologs are also found in eukaryotic systems where CHS may stabilize membrane-protein assemblies (Li, 2022 ▸), although bacterial membranes themselves do not contain cholesterol. Following solubilization, all samples and buffers were supplemented with 0.05%(*w*/*v*) DDM and 0.005%(*w*/*v*) CHS. This preparation yielded grids with significantly reduced aggregation and well dispersed particles (Supplementary Fig. S4*c*). A total of 7506 micrographs were collected for this data set and processed in *cryoSPARC*. Following multiple rounds of 2D classification, 185 213 particles were selected (Supplementary Fig. S4*d*).

Based on the high similarity observed in the 2D class averages, micrographs from this preparation and the previous CHAPS-containing sample were combined after patch CTF estimation. The combined data set was then used to select high-quality trimeric and tetrameric particle classes for subsequent 3D reconstruction.

### 3.4. Cryo-EM analysis and failure to identify the FtsH–YidC complex

Cryo-EM image processing was performed using *cryo­SPARC* (Punjani *et al.*, 2017 ▸). Movies from both data sets were initially processed independently (Supplementary Fig. 4). Patch motion correction and patch CTF estimation (Downing & Glaeser, 2008 ▸; Punjani *et al.*, 2017 ▸) were followed by micrograph curation and particle picking.

Despite being optimized for structural analysis of the FtsH–YidC complex, the data sets did not yield the expected structure. Only a small number of 2D particles possibly corresponding to the cytoplasmic domain of FtsH were detected (Lee *et al.*, 2011 ▸; Qiao *et al.*, 2022 ▸), and no side views or full-length assemblies of FtsH or the FtsH–YidC complex were identified. The absence of identifiable YidC was also not unexpected due to its small molecular size and lack of prominent features in 2D classes.

To explore this further, template-based particle picking was performed in *RELION* (Scheres, 2012 ▸) using the deposited FtsH map (EMDB entry EMD-32524; Qiao *et al.*, 2022 ▸), as well as 2D class average-based picking in *cryoSPARC* (Punjani *et al.*, 2017 ▸). Both approaches failed to retrieve sufficient numbers of FtsH-related particles, supporting their very low abundance or conformational heterogeneity in the vitrified sample.

Consistent 2D class averages were observed across data sets collected from both DDM–CHAPS and DDM–CHS samples, suggesting the presence of similar co-purified species under both detergent conditions.

Unexpectedly, the presence of ArnA and AcrB was first identified through *ab initio* 3D reconstruction, which revealed distinct features resembling known structures of these proteins. This prompted a closer inspection of the 2D class averages, where consistent features supporting their identity were observed across both data sets. Because these proteins were not visible on SDS–PAGE gels and were not the intended targets of the experiment, we retrospectively examined previously acquired MS data, which confirmed the presence of both proteins. The convergence of data from 3D reconstruction, 2D class features and proteomics provided strong evidence for the identity of these particles as ArnA and AcrB.

### 3.5. Off-target structural determination of ArnA and AcrB in the FtsH–YidC cryo-EM data set

Following the identification of ArnA and AcrB particle classes in both data sets, detailed structural analyses were performed to resolve their atomic structures at high resolution. Dominant particle populations corresponding to ArnA and AcrB were consistently identified during data processing, and high-resolution reconstructions of both proteins were obtained. An optimized *cryoSPARC* processing pipeline was established and followed for all subsequent analyses (Fig. 1 ▸). In the 2D class averages, distinct particle classes resembling the cytoplasmic domains of FtsH, GroEL and cytochrome *bo*_3_ oxidase were also observed alongside ArnA and AcrB, but they were not processed further for 3D reconstruction due to low particle numbers and preferred views.

**Figure 2:**
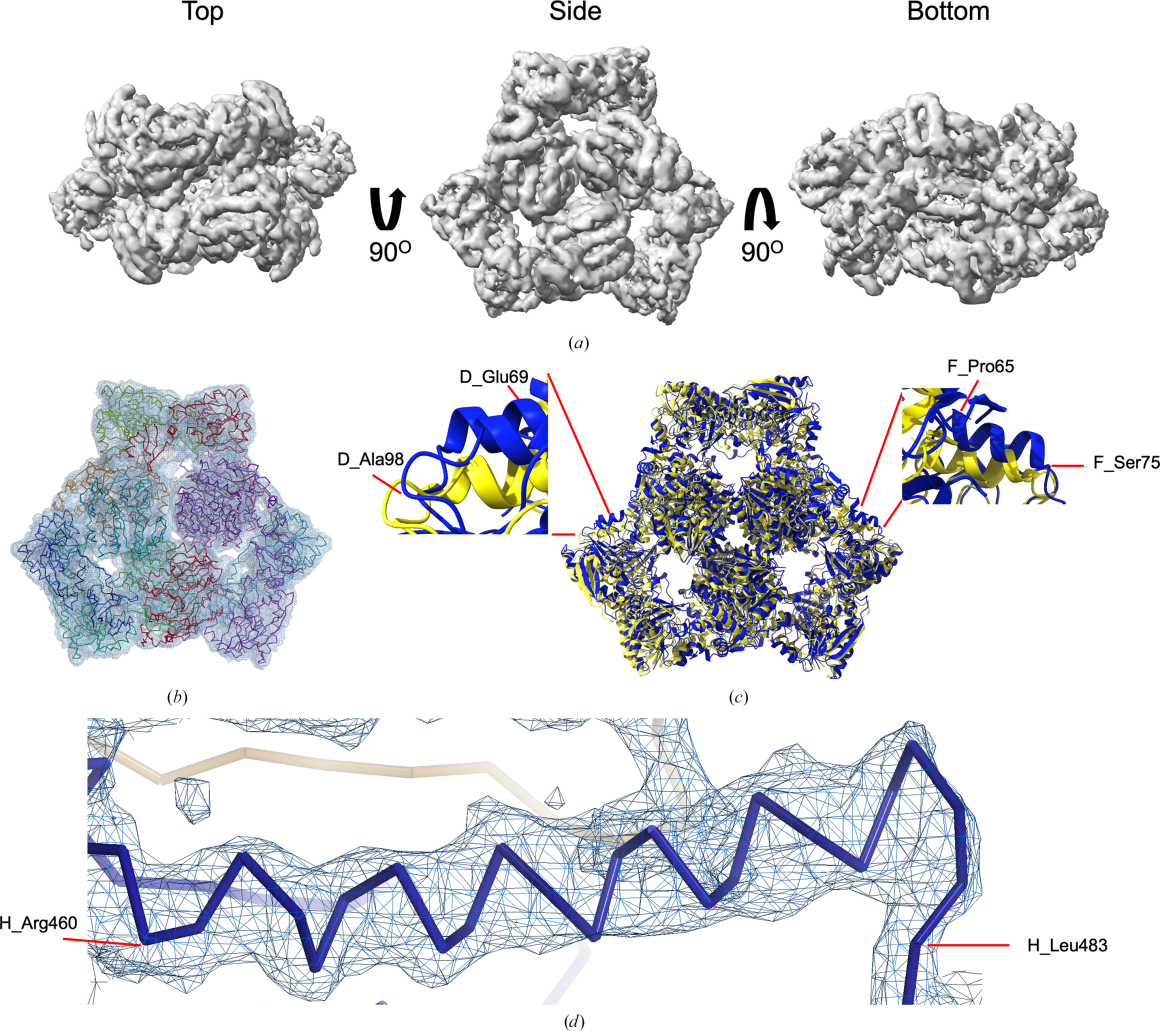
Cryo-EM density map and model fitting of the hexameric ArnA complex. (*a*) Cryo-EM density map of the ArnA hexamer reconstructed at 4.0 Å resolution, shown from three orientations: top, side and bottom views. The map reveals the characteristic two-layered architecture of ArnA and clearly resolved secondary-structure elements. (*b*) Final atomic model of ArnA fitted into the cryo-EM density map. Each of the 12 subunits is displayed in a different color to illustrate the hexameric arrangement. (*c*) Structural alignment of the final cryo-EM model (blue) with the reference crystal structure (yellow) using *ChimeraX*. The alignment yielded an r.m.s.d. of 1.2 Å. Enlarged views show local conformational deviations in loop regions: Glu69–Ala98 in chain *D* (left) and Pro65–Ser75 in chain *F* (right). (*d*) Close-up view showing the model-to-map fit for a peptide segment (Leu483–Arg460). The density mesh is contoured at 1.7σ, showing clear peptide backbone density and supporting accurate model placement.

To achieve high-resolution reconstructions, *ab initio* reconstruction and refinement were performed for ArnA and AcrB. For ArnA, 123 593 particles were processed through heterogeneous and non-uniform refinement, initially using *C*1 symmetry and later *D*3 symmetry. The final reconstruction yielded a map with a global resolution of 4.0 Å, as determined by the FSC_0.143_ criterion (Fig. 2 ▸ and Supplementary Fig. S5).

**Figure 3:**
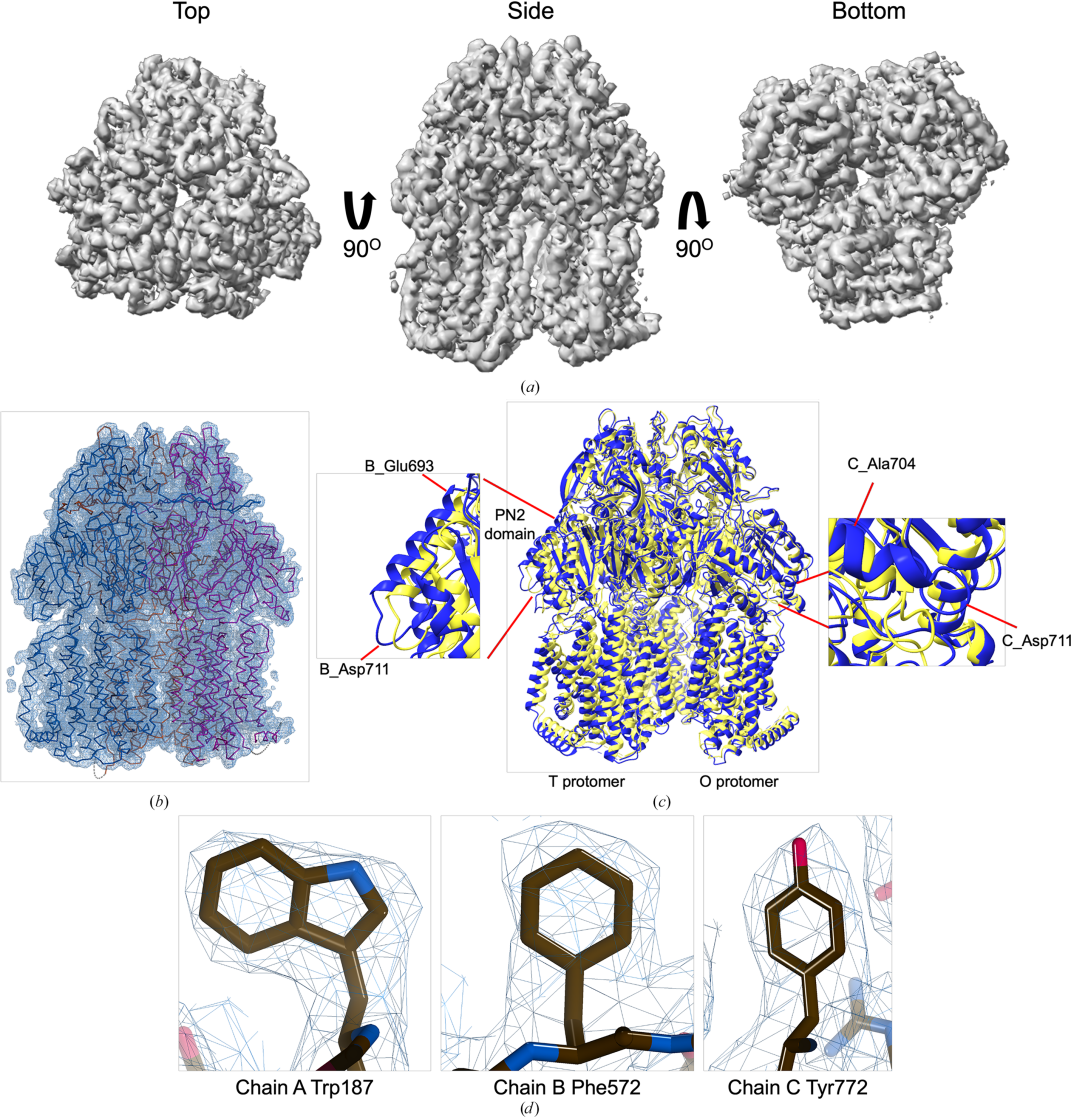
Cryo-EM density map and model fitting of the AcrB trimer. (*a*) Cryo-EM density map of AcrB reconstructed at 2.92 Å resolution, shown from top, side and bottom views. The map reveals clear secondary-structure elements and well resolved transmembrane helices, consistent with the known trimeric architecture. (*b*) Final atomic model of AcrB fitted into the cryo-EM density map. Each protomer is rendered in a different color to highlight the asymmetric trimer organization. (*c*) Structural alignment of the final cryo-EM model (blue) with the reference crystal structure (PDB entry 7rr7, yellow), performed using *ChimeraX*. The r.m.s.d. between the two structures is 1.2 Å. Enlarged views highlight local conformational deviations, including Glu693–Asp711 in protomer *B* (left) and Ala704–Asp711 in protomer *C* (right), located within the PN2 subdomain. (*d*) Close-up view showing the density fit for selected side chains (Trp187 in chain *A*, Phe572 in chain *B*, Tyr772 in chain *C*). The mesh is contoured at 1.6σ in *Coot*, showing well defined density for aromatic residues.

For AcrB, 104 054 particles were refined using *C*1 symmetry, followed by *C*3 symmetry in the final non-uniform refinement step, resulting in a cryo-EM map with a global resolution of 2.92 Å, as determined by the FSC_0.143_ criterion (Fig. 3 ▸ and Supplementary Fig. S6). Global CTF refinement and *DeepEMhancer *post-processing were applied. Handedness correction was performed using *cryoSPARC* volume tools. Local resolution estimation and directional FSC analysis were performed using *Phenix*, confirming moderate anisotropy and high overall map quality for both the ArnA and AcrB reconstructions (Fig. 1 ▸).

### 3.6. High-resolution cryo-EM structure and model validation of hexameric ArnA

A structural model of ArnA was built using PDB entry 6pih as a starting model by molecular replacement using *MOLREP* (Vagin & Teplyakov, 2010 ▸) within the *CCP-EM* interface (Burnley *et al.*, 2017 ▸; Fig. 2 ▸*a*). Refinement was performed using *phenix_real_space_refine*, followed by optimization in *REFMAC* and final manuel adjustments in *Coot* to complete the atomic model (Fig. 2 ▸*b*). The final model was validated using *Phenix* tools, which included both geometry and density correlation analyses (Supplementary Fig. S5*a*).

Local resolution estimation using *Phenix* revealed a range between 3.0 and 5.0 Å across the ArnA complex, with clear secondary-structure elements (Supplementary Fig. S5*b*). The model displayed good agreement with the density (Supplementary Fig. S5*c*), supported by a model-to-map correlation coefficient (CC_mask_) of 0.62 using *Phenix*. The map–model FSC at 0.5 reaches a resolution of 7.5 Å. The overall cryo-EM map resolution, based on the FSC_0.143_ cutoff, was 4.0 Å (Supplementary Fig. S5*a*). The final atomic model comprised 12 chains and 3726 residues, reflecting the hexameric organization of ArnA. Geometry validation indicated a *MolProbity* score of 2.36, with a clashscore of 19.09. Ramachandran analysis showed 95.4% of residues in favored regions and only 0.03% outliers (Table 2 ▸).

To assess structural accuracy, the ArnA cryo-EM model was aligned with a previously determined structure (PDB entry 6pih; Yang *et al.*, 2019 ▸) using *ChimeraX* (Meng *et al.*, 2023 ▸) (Fig. 2 ▸*c*). Alignment with PDB entry 6pih (Yang *et al.*, 2019 ▸), which was used as the starting model for model building, yielded an r.m.s.d. of 1.2 Å over 183 pruned atom pairs and 2.7 Å across all 330 atom pairs, indicating overall consistency between the refined model and its initial template. To provide an additional benchmark, the model was also aligned with PDB entry 4wkg (2.7 Å resolution; Fischer *et al.*, 2015 ▸), the highest-resolution X-ray structure of ArnA available in the PDB. This alignment resulted in an r.m.s.d. of 1.219 Å over 193 pruned atom pairs and 2.487 Å across all 329 C^α^-atom pairs, indicating a high degree of structural similarity. These deviations may reflect conformational flexibility, particularly in loop regions, and are consistent with differences in resolution, oligomeric state or experimental conditions between the cryo-EM and crystallographic structures.

In terms of domain organization, ArnA is a bifunctional cytoplasmic enzyme composed of two catalytic domains: an N-terminal transformylase domain, which catalyzes the formylation of UDP-4-amino-4-deoxy-l-arabinose (UDP-Ara4N), and a C-terminal dehydrogenase domain, which catalyzes the nicotinamide adenine dinucleotide, oxidized form (NAD^+^)-dependent decarboxylation of UDP-glucuronic acid (Fischer *et al.*, 2015 ▸; Gatzeva-Topalova *et al.*, 2005 ▸; Yang *et al.*, 2019 ▸). These domains are connected by a flexible linker and act sequentially in the Ara4N biosynthesis pathway (Fischer *et al.*, 2015 ▸).

Despite the preservation of the global fold, structural comparison revealed several conformational differences relative to the reference model (PDB entry 6pih). Notably, loop regions within the transformylase domain, including residues Glu69–Ala98 in chain *D* and Pro65–Ser75 in chain *F*, exhibited clear deviations (Fig. 2 ▸*c*). Additional variations were observed in other surface-exposed loops, suggesting local flexibility or alternative domain orientations that may be influenced by differences in sample conditions or oligomeric assembly. A previous study has shown that ArnA can adopt multiple oligomeric forms, including hexamers and tetramers, and undergoes conformational changes upon substrate binding (Yang *et al.*, 2019 ▸). In particular, regions near residues 500–509 and 605–616 have been reported to shift in response to UDP-glucuronic acid, facilitating NAD^+^ coordination (Gatzeva-Topalova *et al.*, 2005 ▸).

These observations indicate that the conformational differences identified in the current model, especially within the transformylase domain, may be functionally relevant and reflect the dynamic behavior of ArnA. In addition, the peptide chain of the ArnA model matches the experimental density map well (Fig. 2 ▸*d*).

This analysis provides the highest-resolution cryo-EM reconstruction of ArnA (4.0 Å) currently available in the Electron Microscopy Data Bank (EMDB) and PDB, with earlier models such as PDB entry 6pih reported at 6.6 Å resolution (Yang *et al.*, 2019 ▸).

### 3.7. Structural refinement and validation of AcrB at near-atomic resolution

Model building for AcrB was carried out using the crystal structure PDB entry 7rr7 as the initial template (Trinh *et al.*, 2023 ▸). The model was fitted into the 2.92 Å resolution cryo-EM map through molecular replacement using *MOLREP* within the *CCP-EM* interface (Fig. 3 ▸*a*). Refinement was performed using *Phenix* real-space refinement, followed by optimization in *REFMAC* and final adjustments in *Coot* to complete the atomic model (Fig. 3 ▸*b*).

The final AcrB model consisted of three chains, 3059 residues and 23 153 atoms. Model-validation metrics indicated high stereochemical quality, with a *MolProbity* score of 1.95, a clashscore of 10.36 and 97.2% of residues located in favored Ramachandran regions (Table 2 ▸). Notably, no outliers were observed. The model-to-map correlation coefficient (CC_mask_) was 0.73 (Table 2 ▸). The global cryo-EM map resolution, calculated using the FSC_0.143_ criterion, was 2.92 Å (Supplementary Fig. S6*a*). The map–model FSC at 0.5 reaches a resolution of 3.7 Å (Supplementary Fig. S6*c*). These values collectively reflect a well refined and accurate model, supported by clearly resolved side-chain features throughout all domains (Figs. 3 ▸*b* and 3 ▸*d*).

To evaluate the accuracy of the refined model, the cryo-EM structure of AcrB was compared with the high-resolution crystal structure (PDB entry 7rr7; Trinh *et al.*, 2023 ▸). Structural alignment confirmed that the overall trimeric fold was preserved, with an r.m.s.d. of 1.2 Å across 759 atom pairs (Fig. 3 ▸*c*). Structurally, AcrB functions as a homotrimeric multidrug efflux pump that undergoes a conformational cycling mechanism, with each protomer adopting one of three alternating states: access (loose), binding (tight) or extrusion (open) (Murakami *et al.*, 2002 ▸; Seeger *et al.*, 2006 ▸). This asymmetric arrangement enables unidirectional substrate transport. In the present model, the trimeric architecture and domain organization are consistent with this established mechanism.

While the global fold aligns well with the reference structure, local differences were identified, particularly in flexible loop regions and surface-exposed side chains. Notable deviations were observed between Asp711 and Glu693, which reside within the PN2 subdomain of the T protomer (Seeger *et al.*, 2006 ▸; Fig. 3 ▸*c*). This region has been implicated in substrate accommodation and undergoes conformational shifts during the efflux (Murakami *et al.*, 2002 ▸; Ababou & Koronakis, 2016 ▸). Furthermore, although the gate loop (residues 615–620) was not directly analyzed in this study, previous structural and mutagenesis studies have shown that this segment modulates the progression of substrates between the proximal and distal binding pockets, especially in the case of large molecules such as erythromycin (Ababou & Koronakis, 2016 ▸).

The refined model also shows reasonable side-chain-level agreement with the cryo-EM density in well ordered regions (Fig. 3 ▸*d*), further supporting its accuracy. The data confirm that AcrB at 2.92 Å (Supplementary Fig. S5*a*), although not the intended target of structural analysis, was abundantly and stably present in the FtsH–YidC sample, enabling near-atomic characterization via single-particle cryo-EM (Fig. 1 ▸).

## 4. Discussion

### 4.1. Aggregation behavior observed in cryo-EM compared with negative-stain EM

Although the same protein samples were used, a clear difference in particle distribution was observed between cryo-EM and negative-stain EM (NS-EM). NS-EM micrographs showed well dispersed particles and no aggregation. In contrast, aggregation was present in cryo-EM grids, depending on the sample-preparation conditions. In the first cryo-EM screening, DDM was used as the main detergent, and the sample was cross-linked with 0.25 m*M* DSP. CHAPS was not included at this stage, and aggregated particles were evident (Supplementary Fig. S4).

In the second and third screenings, 0.4%(*w*/*v*) CHAPS was added to the 1 m*M* DSP-cross-linked sample 10 min before vitrification. Under these conditions the particle distribution improved and no aggregation was observed. CHAPS, a zwitterionic detergent, has been reported to reduce surface tension and support membrane-protein stabilization during freezing steps (Li, 2022 ▸; Kampjut *et al.*, 2021 ▸; Egri *et al.*, 2023 ▸). Its use just before vitrification may have helped to maintain sample quality on the grid without affecting detergent micelle formation during purification.

In a separate experiment, CHS was added together with DDM during solubilization and purification. CHS mimics native cholesterol and has been shown to enhance membrane-protein integrity in structural studies (Li, 2022 ▸; Miyata *et al.*, 2025 ▸). Among all conditions tested, the CHS-containing sample displayed the lowest level of aggregation, and the grid quality was notably higher (Supplementary Fig. S3).

### 4.2. Absence of FtsH–YidC complex reconstruction in cryo-EM analysis

The aim of our work was to determine the structure of the FtsH–YidC complex using single-particle cryo-EM. However, neither 2D class averages nor 3D classes resembling FtsH, YidC or FtsH–HflKC assemblies were observed in the data sets (Ghanbarpour *et al.*, 2025 ▸; Kedrov *et al.*, 2016 ▸; Kumazaki *et al.*, 2014 ▸; Langklotz *et al.*, 2012 ▸; Lee *et al.*, 2011 ▸; Ma *et al.*, 2022 ▸; Qiao *et al.*, 2022 ▸; Tanaka *et al.*, 2018 ▸). Among the expected components, only 2D class averages resembling the cytoplasmic AAA+ domain of FtsH were detected (Fig. 1 ▸). Side views corresponding to full-length FtsH, *i.e.* including the transmembrane region, were not observed (Carvalho *et al.*, 2021 ▸; Ghanbarpour *et al.*, 2025 ▸; Liu *et al.*, 2022 ▸; Ma *et al.*, 2022 ▸; Qiao *et al.*, 2022 ▸). The transmembrane and cytoplasmic regions of FtsH are known to adopt variable conformations (Carvalho *et al.*, 2021 ▸; Liu *et al.*, 2022 ▸), and these dynamics likely interfered with consistent alignment and classification during image processing, yielding no side views of FtsH. Additionally, our recent biochemical evidence indicates that the interaction between FtsH and YidC is transient rather than stable, further reducing the likelihood of capturing a complete and well defined FtsH–YidC complex in vitrified samples (Caliseki, Zorman *et al.*, 2025 ▸).

No particle classes corresponding to full-length YidC were identified either. This may be due to its relatively small size (∼61 kDa), and its flexibility between the transmembrane and periplasmic domains, which limit its visibility and alignment in single-particle cryo-EM (Kumazaki *et al.*, 2014 ▸; Tanaka *et al.*, 2018 ▸). Despite strong biochemical evidence for the FtsH–YidC interaction (Supplementary Figs. S1–S3; Caliseki, Zorman *et al.*, 2025 ▸), we could not visualize the complex in single-particle cryo-EM.

### 4.3. High-resolution reconstruction of the off-target proteins ArnA and AcrB

While the targeted FtsH–YidC complex could not be resolved in cryo-EM data sets, cryo-EM models of two abundant off-target proteins, ArnA and AcrB, were successfully reconstructed at high resolution. ArnA, a cytoplasmic hexamer involved in lipid A modification, was reconstructed at 4.0 Å resolution, and AcrB, a trimeric inner membrane transporter of the AcrAB–TolC efflux system, was resolved at 2.92 Å resolution. Both proteins are known to form stable oligomeric assemblies and are frequently reported as co-purifying species in protein preparations (Andersen *et al.*, 2013 ▸; Glover *et al.*, 2011 ▸; Veesler *et al.*, 2008 ▸).

Mass-spectrometry analysis confirmed the presence of ArnA and AcrB in samples purified using both Ni–NTA and Strep-Tactin affinity chromatography (Supplementary Table S1; Caliseki, Zorman *et al.*, 2025 ▸). However, these proteins were not clearly detected in SDS–PAGE analyses of SEC fractions, indicating that their concentrations at this stage were below the sensitivity of gel-based visualization. This difference between MS and SDS–PAGE results suggests that while their overall abundance was low, their particle abundance in cryo-grids, structural rigidity and symmetry were sufficient to enable efficient classification and reconstruction. In addition, their particle diameter is similar to the cytoplasmic domain of FtsH (14 Å).

ArnA is a cytoplasmic protein and would not be expected to co-enrich with membrane fractions in ultracentrifugation. Its identification in detergent-solubilized membrane-protein samples may indicate a possible interaction, directly or indirectly, with membrane components such as FtsH or YidC. Although ArnA was not detectable by Coomassie-stained SDS–PAGE, single-particle cryo-EM analysis yielded a 4.0 Å resolution reconstruction, which currently constitutes the highest resolution cryo-EM structure of this protein available in both the EMDB and PDB.

Similarly, AcrB was consistently identified in mass spectrometry across different purification steps (Supplementary Table S1; Caliseki, Zorman *et al.*, 2025 ▸), reflecting its structural integrity and biochemical stability in detergent micelles. These properties likely facilitated its retention through the cryo-EM grid-preparation and image-processing workflow facilitated by accurate particle alignment (Su *et al.*, 2021 ▸).

### 4.4. Cryo-EM reveals co-purified components linked to proteostasis and aerobic metabolism

In this study, FtsH, YidC and HflKC were co-overexpressed; however, purification predominantly yielded FtsH and YidC, while HflKC was not reliably detected. Two factors may explain the absence of HflKC. Firstly, the mutually exclusive interaction between HflKC and YidC for FtsH binding could lead to competitive exclusion of HflKC. Secondly, previous transcriptomic studies have shown that depletion of YidC leads to increased expression of HflK and HflC (Wickström *et al.*, 2011 ▸), raising the possibility that YidC overexpression may inversely affect HflKC levels. However, further experiments would be required to directly assess HflKC expression levels or membrane incorporation under these conditions.

Cryo-EM 2D class averages revealed particles corresponding to cytochrome *bo*_3_ oxidase, a key component of the aerobic respiratory chain that requires YidC for membrane insertion (Celebi *et al.*, 2006 ▸; Du Plessis *et al.*, 2006 ▸). This observation may reflect a functional association with YidC, consistent with its known role in respiratory complex biogenesis. Alternatively, the presence of cytochrome *bo*_3_ may be influenced by the apparent absence or very low abundance of HflKC, which remained barely detectable in the purified sample despite being co-overexpressed (Caliseki, Zorman *et al.*, 2025 ▸). HflKC has been implicated in the regulation of cytochrome oxidase expression and aerobic metabolism (Pérez-López *et al.*, 2025 ▸). Although this remains speculative in the context of the present study, the findings raise the possibility that HflKC levels may influence the abundance or stability of cytochrome *bo*_3_ under stress conditions.

Finally, the failure to observe an intact FtsH–YidC complex, along with the enrichment of cytochrome *bo*_3_ (Borisov *et al.*, 2021 ▸) and GroEL (Fourie & Wilson, 2020 ▸), likely reflects the dynamic and transient nature of YidC interactions, and the interactome being shaped by the cellular metabolic state and proteostasis demands. The complexity of these interactions is further reflected in the difficulty of resolving intact YidC complexes in our cryo-EM data sets. However, recent advances, such as the BaR method, have demonstrated the capability of cryo-EM to resolve multiple protein structures from heterogeneous samples (Su *et al.*, 2021 ▸). The BaR approach highlights the remarkable power of cryo-EM to capture diverse structural states within a single data set, even from complex mixtures. In our study, this capability allowed us to resolve the structures of ArnA and AcrB while also identifying 2D class averages resembling GroEL and cytochrome *bo*_3_, further emphasizing the dynamic nature of the protein mixtures.

In summary, our findings underscore the complexity of cryo-EM data sets derived from membrane-protein complexes and highlight the importance of integrating structural and biochemical data with mass spectrometry to accurately interpret the data.

## 5. Conclusion

This study highlights the complexity of transient membrane-protein interactions, such as that of YidC and FtsH involved in membrane-protein homeostasis, and the associated challenges to obtain structural and functional insights into these protein complexes by cryo-EM. While the FtsH–YidC complex could not be reconstructed, high-resolution structures of off-target proteins, such as ArnA and AcrB, were successfully obtained, providing valuable structural insights into these components. The enrichment of co-purified proteins in cryo-EM 2D class averages, such as GroEL and cytochrome *bo*_3_ (also identified in mass spectrometry), further illustrates the complexity of the task. Our findings emphasize the importance of combining structural and biochemical approaches and mass spectrometry to gain a comprehensive understanding of membrane-protein function and regulation, particularly in the context of stress-induced cellular responses.

## Supplementary Material

PDB reference: cryo-EM structure of hexameric ArnA, 9v5h

PDB reference: cryo-EM structure of trimeric AcrB, 9v5r

EMDB reference: cryo-EM structure of hexameric ArnA, EMD-64789

EMDB reference: cryo-EM structure of trimeric AcrB, EMD-64793

Supplementary Figures and Table. DOI: 10.1107/S2059798325007089/jb5069sup1.pdf

## Figures and Tables

**Table 1:** Cryo-EM data collection and initial data processing

	DDM–CHAPS sample	DDM–CHS sample
Voltage (kV)	200	200
Pixel size (Å per pixel)	1.05 (0.525)	1.05 (0.525)
Nominal magnification	130000	130000
Exposure (e^−^ Å^−2^)	58.2	60.6
Frames per exposure	60	60
Defocus range (µm)	−0.7 to −2.2	−0.7 to −2.2
No. of collected micrographs	3908	7506
Micrographs in combined data sets	10141	

**Table 2:** Cryo-EM data processing and protein modeling for ArnA and AcrB

	ArnA	AcrB
Final particle images	74212	77800
Symmetry imposed	*D*3	*C*3
Resolution at FSC = 0.143 (Å)	4.0	2.92
Map sharpening	*DeepEMhancer*	*DeepEMhancer*
Reference PDB code	6pih	7rr7
No. of chains	12	3
No. of residues	3726	3059
No. of atoms	29508	23153
*MolProbity* score	2.36	1.95
Clashscore	19.09	10.36
Ramachandran favored (%)	95.4	97.2
Ramachandran outliers (%)	0.03	0.0
Model-to-map correlation (CC_mask_)	0.62	0.73
Model-to-map FSC = 0.5 (Å)	4.9	3.7
R.m.s.d. (pruned atom pairs) (Å)	1.2 (183 pairs)	1.2 (759 pairs)
R.m.s.d. (all atom pairs) (Å)	2.7 (330 pairs)	2.0 (1025 pairs)

## Data Availability

All data needed to evaluate the conclusions in the paper are present in the paper and/or the supporting information. All data sets generated during the current study have been deposited in the Electron Microscopy Data Bank (EMDB) as entries EMD-64789 (ArnA) and EMD-64793 (AcrB) and in the Protein Data Bank (PDB) as PDB entries 9v5h (ArnA) and 9v5r (AcrB).

## References

[bb1] Ababou, A. & Koronakis, V. (2016). *PLoS One*, **11**, e0159154. 10.1371/journal.pone.0159154 PMC4942123 27403665

[bb2] Afonine, P. V., Klaholz, B. P., Moriarty, N. W., Poon, B. K., Sobolev, O. V., Terwilliger, T. C., Adams, P. D. & Urzhumtsev, A. (2018). *Acta Cryst.* D**74**, 814–840. 10.1107/S2059798318009324 PMC6130467 30198894

[bb3] Agirre, J., Atanasova, M., Bagdonas, H., Ballard, C. B., Baslé, A., Beilsten-Edmands, J., Borges, R. J., Brown, D. G., Burgos-Mármol, J. J., Berrisford, J. M., Bond, P. S., Caballero, I., Catapano, L., Chojnowski, G., Cook, A. G., Cowtan, K. D., Croll, T. I., Debreczeni, J. É., Devenish, N. E., Dodson, E. J., Drevon, T. R., Emsley, P., Evans, G., Evans, P. R., Fando, M., Foadi, J., Fuentes-Montero, L., Garman, E. F., Gerstel, M., Gildea, R. J., Hatti, K., Hekkelman, M. L., Heuser, P., Hoh, S. W., Hough, M. A., Jenkins, H. T., Jiménez, E., Joosten, R. P., Keegan, R. M., Keep, N., Krissinel, E. B., Kolenko, P., Kovalevskiy, O., Lamzin, V. S., Lawson, D. M., Lebedev, A. A., Leslie, A. G. W., Lohkamp, B., Long, F., Malý, M., McCoy, A. J., McNicholas, S. J., Medina, A., Millán, C., Murray, J. W., Murshudov, G. N., Nicholls, R. A., Noble, M. E. M., Oeffner, R., Pannu, N. S., Parkhurst, J. M., Pearce, N., Pereira, J., Perrakis, A., Powell, H. R., Read, R. J., Rigden, D. J., Rochira, W., Sammito, M., Sánchez Rodríguez, F., Sheldrick, G. M., Shelley, K. L., Simkovic, F., Simpkin, A. J., Skubak, P., Sobolev, E., Steiner, R. A., Stevenson, K., Tews, I., Thomas, J. M. H., Thorn, A., Valls, J. T., Uski, V., Usón, I., Vagin, A., Velankar, S., Vollmar, M., Walden, H., Waterman, D., Wilson, K. S., Winn, M. D., Winter, G., Wojdyr, M. & Yamashita, K. (2023). *Acta Cryst.* D**79**, 449–461.

[bb4] Akiyama, Y. (2002). *Proc. Natl Acad. Sci. USA*, **99**, 8066–8071. 10.1073/pnas.122616899 PMC123021 12034886

[bb5] Akiyama, Y. (2009). *J. Biochem.* **146**, 449–454. 10.1093/jb/mvp071 19454621

[bb6] Akkulak, H., İnce, H. K., Goc, G., Lebrilla, C. B., Kabasakal, B. V. & Ozcan, S. (2024). *Int. J. Biol. Macromol.* **269**, 131923. 10.1016/j.ijbiomac.2024.131923 38697437

[bb7] Andersen, K. R., Leksa, N. C. & Schwartz, T. U. (2013). *Proteins*, **81**, 1857–1861. 10.1002/prot.24364 PMC4086167 23852738

[bb8] Beck, K., Eisner, G., Trescher, D., Dalbey, R. E., Brunner, J. & Müller, M. (2001). *EMBO Rep.* **2**, 709–714. 10.1093/embo-reports/kve154 PMC1083991 11463745

[bb9] Bieniossek, C., Schalch, T., Bumann, M., Meister, M., Meier, R. & Baumann, U. (2006). *Proc. Natl Acad. Sci. USA*, **103**, 3066–3071. 10.1073/pnas.0600031103 PMC1413944 16484367

[bb10] Bolanos-Garcia, V. M. & Davies, O. R. (2006). *Biochim. Biophys. Acta*, **1760**, 1304–1313. 10.1016/j.bbagen.2006.03.027 16814929

[bb11] Borisov, V. B., Siletsky, S. A., Paiardini, A., Hoogewijs, D., Forte, E., Giuffrè, A. & Poole, R. K. (2021). *Antioxid. Redox Signal.* **34**, 1280–1318. 10.1089/ars.2020.8039 PMC8112716 32924537

[bb12] Botte, M., Zaccai, N. R., Nijeholt, J. L. À., Martin, R., Knoops, K., Papai, G., Zou, J., Deniaud, A., Karuppasamy, M., Jiang, Q., Roy, A. S., Schulten, K., Schultz, P., Rappsilber, J., Zaccai, G., Berger, I., Collinson, I. & Schaffitzel, C. (2016). *Sci. Rep.* **6**, 38399. 10.1038/srep38399 PMC5141469 27924919

[bb13] Burnley, T., Palmer, C. M. & Winn, M. (2017). *Acta Cryst.* D**73**, 469–477. 10.1107/S2059798317007859 PMC5458488 28580908

[bb15] Caliseki, M., Schaffitzel, C. & Kabasakal, B. V. (2025). *Biochim. Biophys. Acta*, **1872**, 119956. 10.1016/j.bbamcr.2025.119956 PMC7618853 40221051

[bb14] Caliseki, M., Zorman, S., Schaffitzel, C. & Kabasakal, B. V. (2025). *SSRN*, https://dx.doi.org/10.2139/ssrn.5200441.

[bb16] Carvalho, V., Prabudiansyah, I., Kovacik, L., Chami, M., Kieffer, R., van der Valk, R., de Lange, N., Engel, A. & Aubin-Tam, M.-E. (2021). *J. Biol. Chem.* **296**, 100029. 10.1074/jbc.RA120.014739 PMC7949044 33154162

[bb17] Celebi, N., Yi, L. J., Facey, S., Kuhn, A. & Dalbey, R. E. (2006). *J. Mol. Biol.* **357**, 1428–1436. 10.1016/j.jmb.2006.01.030 16488430

[bb18] Dalbey, R. E., Kuhn, A., Zhu, L. & Kiefer, D. (2014). *Biochim. Biophys. Acta*, **1843**, 1489–1496. 10.1016/j.bbamcr.2013.12.022 24418623

[bb19] Dalbey, R. E., Wang, P. & van Dijl, J. M. (2012). *Microbiol. Mol. Biol. Rev.* **76**, 311–330. 10.1128/MMBR.05019-11 PMC3372248 22688815

[bb20] Downing, K. H. & Glaeser, R. M. (2008). *Ultramicroscopy*, **108**, 921–928. 10.1016/j.ultramic.2008.03.004 PMC2694513 18508199

[bb21] du Plessis, D. J. F., Nouwen, N. & Driessen, A. J. M. (2006). *J. Biol. Chem.* **281**, 12248–12252. 10.1074/jbc.M600048200 16513637

[bb22] Egri, S. B., Wang, X., Díaz-Salinas, M. A., Luban, J., Dudkina, N. V., Munro, J. B. & Shen, K. (2023). *Nat. Commun.* **14**, 2527. 10.1038/s41467-023-38251-9 PMC10154187 37137903

[bb23] Emsley, P., Lohkamp, B., Scott, W. G. & Cowtan, K. (2010). *Acta Cryst.* D**66**, 486–501. 10.1107/S0907444910007493 PMC2852313 20383002

[bb24] Ero, R., Qiao, Z., Tan, K. A. & Gao, Y.-G. (2024). *Biochem. Soc. Trans.* **52**, 2077–2086. 10.1042/BST20231250 39417347

[bb25] Fischer, U., Hertlein, S. & Grimm, C. (2015). *Acta Cryst.* D**71**, 687–696. 10.1107/S1399004714026686 25760615

[bb26] Fourie, K. R. & Wilson, H. L. (2020). *Vaccines*, **8**, 773. 10.3390/vaccines8040773 PMC7767184 33348708

[bb27] Fujita, J., Makino, F., Asahara, H., Moriguchi, M., Kumano, S., Anzai, I., Kishikawa, J., Matsuura, Y., Kato, T., Namba, K. & Inoue, T. (2023). *Sci. Rep.* **13**, 2279. 10.1038/s41598-023-29396-0 PMC9908306 36755111

[bb28] Gatzeva-Topalova, P. Z., May, A. P. & Sousa, M. C. (2005). *Structure*, **13**, 929–942. 10.1016/j.str.2005.03.018 PMC2997725 15939024

[bb29] Ghanbarpour, A., Telusma, B., Powell, B. M., Zhang, J. J., Bolstad, I., Vargas, C., Keller, S., Baker, T. A., Sauer, R. T. & Davis, J. H. (2025). *EMBO J.* **44**, 2501–2513. 10.1038/s44318-025-00408-1 PMC12048511 40082723

[bb30] Glover, C. A. P., Postis, V. L. G., Charalambous, K., Tzokov, S. B., Booth, W. I., Deacon, S. E., Wallace, B. A., Baldwin, S. A. & Bullough, P. A. (2011). *J. Struct. Biol.* **176**, 419–424. 10.1016/j.jsb.2011.09.005 21964467

[bb31] Houben, E. N. G., Scotti, P. A., Valent, Q. A., Brunner, J., de Gier, J. L., Oudega, B. & Luirink, J. (2000). *FEBS Lett.* **476**, 229–233. 10.1016/s0014-5793(00)01735-x 10913619

[bb32] Ito, K. & Akiyama, Y. (2005). *Annu. Rev. Microbiol.* **59**, 211–231. 10.1146/annurev.micro.59.030804.121316 15910274

[bb33] Kampjut, D., Steiner, J. & Sazanov, L. A. (2021). *iScience*, **24**, 102139. 10.1016/j.isci.2021.102139 PMC7900225 33665558

[bb34] Kedrov, A., Wickles, S., Crevenna, A. H., van der Sluis, E. O., Buschauer, R., Berninghausen, O., Lamb, D. C. & Beckmann, R. (2016). *Cell. Rep.* **17**, 2943–2954. 10.1016/j.celrep.2016.11.059 PMC5186731 27974208

[bb35] Kihara, A., Akiyama, Y. & Ito, K. (1998). *J. Mol. Biol.* **279**, 175–188. 10.1006/jmbi.1998.1781 9636708

[bb36] Komar, J., Alvira, S., Schulze, R. J., Martin, R., Lycklama a Nijeholt, J. A., Lee, S. C., Dafforn, T. R., Deckers-Hebestreit, G., Berger, I., Schaffitzel, C. & Collinson, I. (2016). *Biochem. J.* **473**, 3341–3354. 10.1042/BCJ20160545 PMC5095914 27435098

[bb37] Kumazaki, K., Kishimoto, T., Furukawa, A., Mori, H., Tanaka, Y., Dohmae, N., Ishitani, R., Tsukazaki, T. & Nureki, O. (2014). *Sci. Rep.* **4**, 7299. 10.1038/srep07299 PMC4252904 25466392

[bb39] Langklotz, S., Baumann, U. & Narberhaus, F. (2012). *Biochim. Biophys. Acta*, **1823**, 40–48. 10.1016/j.bbamcr.2011.08.015 21925212

[bb40] Lee, S., Augustin, S., Tatsuta, T., Gerdes, F., Langer, T. & Tsai, F. T. F. (2011). *J. Biol. Chem.* **286**, 4404–4411. 10.1074/jbc.M110.158741 PMC3039362 21147776

[bb41] Li, S. (2022). *Acta Biochim. Biophys. Sin.* **54**, 1049–1056. 10.3724/abbs.2022088 PMC9828306 35866608

[bb42] Liebschner, D., Afonine, P. V., Baker, M. L., Bunkóczi, G., Chen, V. B., Croll, T. I., Hintze, B., Hung, L.-W., Jain, S., McCoy, A. J., Moriarty, N. W., Oeffner, R. D., Poon, B. K., Prisant, M. G., Read, R. J., Richardson, J. S., Richardson, D. C., Sammito, M. D., Sobolev, O. V., Stockwell, D. H., Terwilliger, T. C., Urzhumtsev, A. G., Videau, L. L., Williams, C. J. & Adams, P. D. (2019). *Acta Cryst.* D**75**, 861–877. 10.1107/S2059798319011471 PMC6778852 31588918

[bb43] Liu, G., Beaton, S. E., Grieve, A. G., Evans, R., Rogers, M., Strisovsky, K., Armstrong, F. A., Freeman, M., Exley, R. M. & Tang, C. M. (2020). *EMBO J.* **39**, e102922. 10.15252/embj.2019102922 PMC7232013 32337752

[bb44] Liu, W., Schoonen, M., Wang, T., McSweeney, S. & Liu, Q. (2022). *Commun. Biol.* **5**, 257. 10.1038/s42003-022-03213-2 PMC8943139 35322207

[bb45] Ma, C., Wang, C., Luo, D., Yan, L., Yang, W., Li, N. & Gao, N. (2022). *Cell Res.* **32**, 176–189. 10.1038/s41422-021-00598-3 PMC8807802 34975153

[bb46] Meng, E. C., Goddard, T. D., Pettersen, E. F., Couch, G. S., Pearson, Z. J., Morris, J. H. & Ferrin, T. E. (2023). *Protein Sci.* **32**, e4792. 10.1002/pro.4792 PMC10588335 37774136

[bb47] Miyata, Y., Takahashi, K., Lee, Y., Sultan, C. S., Kuribayashi, R., Takahashi, M., Hata, K., Bamba, T., Izumi, Y., Liu, K., Uemura, T., Nomura, N., Iwata, S., Nagata, S., Nishizawa, T. & Segawa, K. (2025). *Nat. Struct. Mol. Biol.* **32**, 185–198. 10.1038/s41594-024-01411-6 PMC11753361 39424995

[bb48] Murakami, S., Nakashima, R., Yamashita, E. & Yamaguchi, A. (2002). *Nature*, **419**, 587–593. 10.1038/nature01050 12374972

[bb49] Njenga, R., Boele, J., Öztürk, Y. & Koch, H.-G. (2023). *J. Biol. Chem.* **299**, 105163. 10.1016/j.jbc.2023.105163 PMC10502375 37586589

[bb50] Pérez-López, M. I., Lubrano, P., Angelidou, G., Hoch, S., Glatter, T., Paczia, N., Link, H. & Sourjik, V. (2025). *PLoS Biol.* **23**, e3003077. 10.1371/journal.pbio.3003077 PMC12005517 40193326

[bb51] Petriman, N.-A., Jauss, B., Hufnagel, A., Franz, L., Sachelaru, I., Drepper, F., Warscheid, B. & Koch, H.-G. (2018). *Sci. Rep.* **8**, 578. 10.1038/s41598-017-19019-w PMC5766551 29330529

[bb52] Punjani, A., Rubinstein, J. L., Fleet, D. J. & Brubaker, M. A. (2017). *Nat. Methods*, **14**, 290–296. 10.1038/nmeth.4169 28165473

[bb53] Punjani, A., Zhang, H. & Fleet, D. J. (2020). *Nat. Methods*, **17**, 1214–1221. 10.1038/s41592-020-00990-8 33257830

[bb54] Qiao, Z., Yokoyama, T., Yan, X.-F., Beh, I. T., Shi, J., Basak, S., Akiyama, Y. & Gao, Y.-G. (2022). *Cell. Rep.* **39**, 110890. 10.1016/j.celrep.2022.110890 35649372

[bb55] Saikawa, N., Akiyama, Y. & Ito, K. (2004). *J. Struct. Biol.* **146**, 123–129. 10.1016/j.jsb.2003.09.020 15037243

[bb56] Sanchez-Garcia, R., Gomez-Blanco, J., Cuervo, A., Carazo, J. M., Sorzano, C. O. S. & Vargas, J. (2021). *Commun. Biol.* **4**, 874. 10.1038/s42003-021-02399-1 PMC8282847 34267316

[bb57] Scheres, S. H. W. (2012). *J. Struct. Biol.* **180**, 519–530. 10.1016/j.jsb.2012.09.006 PMC3690530 23000701

[bb58] Schulze, R. J., Komar, J., Botte, M., Allen, W. J., Whitehouse, S., Gold, V. A. M., Lycklama a Nijeholt, J. A., Huard, K., Berger, I., Schaffitzel, C. & Collinson, I. (2014). *Proc. Natl Acad. Sci. USA*, **111**, 4844–4849. 10.1073/pnas.1315901111 PMC3977283 24550475

[bb59] Seeger, M. A., Schiefner, A., Eicher, T., Verrey, F., Diederichs, K. & Pos, K. M. (2006). *Science*, **313**, 1295–1298. 10.1126/science.1131542 16946072

[bb60] Shanmugam, S. K., Backes, N., Chen, Y., Belardo, A., Phillips, G. J. & Dalbey, R. E. (2019). *J. Mol. Biol.* **431**, 1025–1037. 10.1016/j.jmb.2019.01.006 30639187

[bb61] Su, C.-C., Lyu, M., Morgan, C. E., Bolla, J. R., Robinson, C. V. & Yu, E. W. (2021). *Nat. Methods*, **18**, 69–75. 10.1038/s41592-020-01021-2 PMC7808410 33408407

[bb62] Takatsuka, Y., Chen, C. & Nikaido, H. (2010). *Proc. Natl Acad. Sci. USA*, **107**, 6559–6565. 10.1073/pnas.1001460107 PMC2872455 20212112

[bb63] Tanaka, Y., Izumioka, A., Abdul Hamid, A., Fujii, A., Haruyama, T., Furukawa, A. & Tsukazaki, T. (2018). *Biochem. Biophys. Res. Commun.* **505**, 141–145. 10.1016/j.bbrc.2018.09.043 30241934

[bb64] Trinh, T. K. H., Cabezas, A. J., Joshi, S., Catalano, C., Siddique, A. B., Qiu, W., Deshmukh, S., des Georges, A. & Guo, Y. (2023). *Chem. Sci.* **14**, 7310–7326. 10.1039/d3sc01890c PMC10321531 37416719

[bb65] Vagin, A. & Teplyakov, A. (2010). *Acta Cryst.* D**66**, 22–25. 10.1107/S0907444909042589 20057045

[bb66] van Bloois, E., Dekker, H. L., Fröderberg, L., Houben, E. N. G., Urbanus, M. L., de Koster, C. G., de Gier, J.-W. & Luirink, J. (2008). *FEBS Lett.* **582**, 1419–1424. 10.1016/j.febslet.2008.02.082 18387365

[bb38] van der Laan, M., Nouwen, N. P. & Driessen, A. J. (2005). *Curr. Opin. Microbiol.* **8**, 182–187. 10.1016/j.mib.2005.02.004 15802250

[bb67] Veesler, D., Blangy, S., Cambillau, C. & Sciara, G. (2008). *Acta Cryst.* F**64**, 880–885. 10.1107/S1744309108028248 PMC2564894 18931428

[bb68] Watkins, D. W., Williams, S. L. & Collinson, I. (2022). *Microbiology*, **168**, https://doi.org/10.1099/mic.0.001255. 10.1099/mic.0.001255 36260397

[bb69] Wickström, D., Wagner, S., Simonsson, P., Pop, O., Baars, L., Ytterberg, A. J., van Wijk, K. J., Luirink, J. & de Gier, J. L. (2011). *J. Mol. Biol.* **409**, 124–135. 10.1016/j.jmb.2011.03.068 21497606

[bb70] Williams, G. J., Breazeale, S. D., Raetz, C. R. H. & Naismith, J. H. (2005). *J. Biol. Chem.* **280**, 23000–23008. 10.1074/jbc.M501534200 PMC3326539 15809294

[bb71] Yang, M., Chen, Y. S., Ichikawa, M., Calles-Garcia, D., Basu, K., Fakih, R., Bui, K. H. & Gehring, K. (2019). *J. Struct. Biol.* **208**, 43–50. 10.1016/j.jsb.2019.07.009 31344437

