# Supplementary material for: Off-target structural insights: ArnA and AcrB in bacterial membrane-protein cryo-EM analysis
Source: Acta Crystallogr D Struct Biol. 2025 Sep 10;81(Pt 10):545–57. doi: 10.1107/S2059798325007089 (PMC12485490; doi:10.1107/S2059798325007089)
Supplement: Supplementary file 1 [file d-81-00545-sup1.pdf]

Acta Cryst D

STRUCTURAL BIOLOGY

Volume 81 (2025)

Supporting information for article:

Off-target structural insights: ArnA and AcrB in bacterial membrane-protein cryo-EM analysis

Mehmet Caliseki, Ufuk Borucu, Sathish K. N. Yadav, Christiane Schaffitzel and Burak Veli Kabasakal

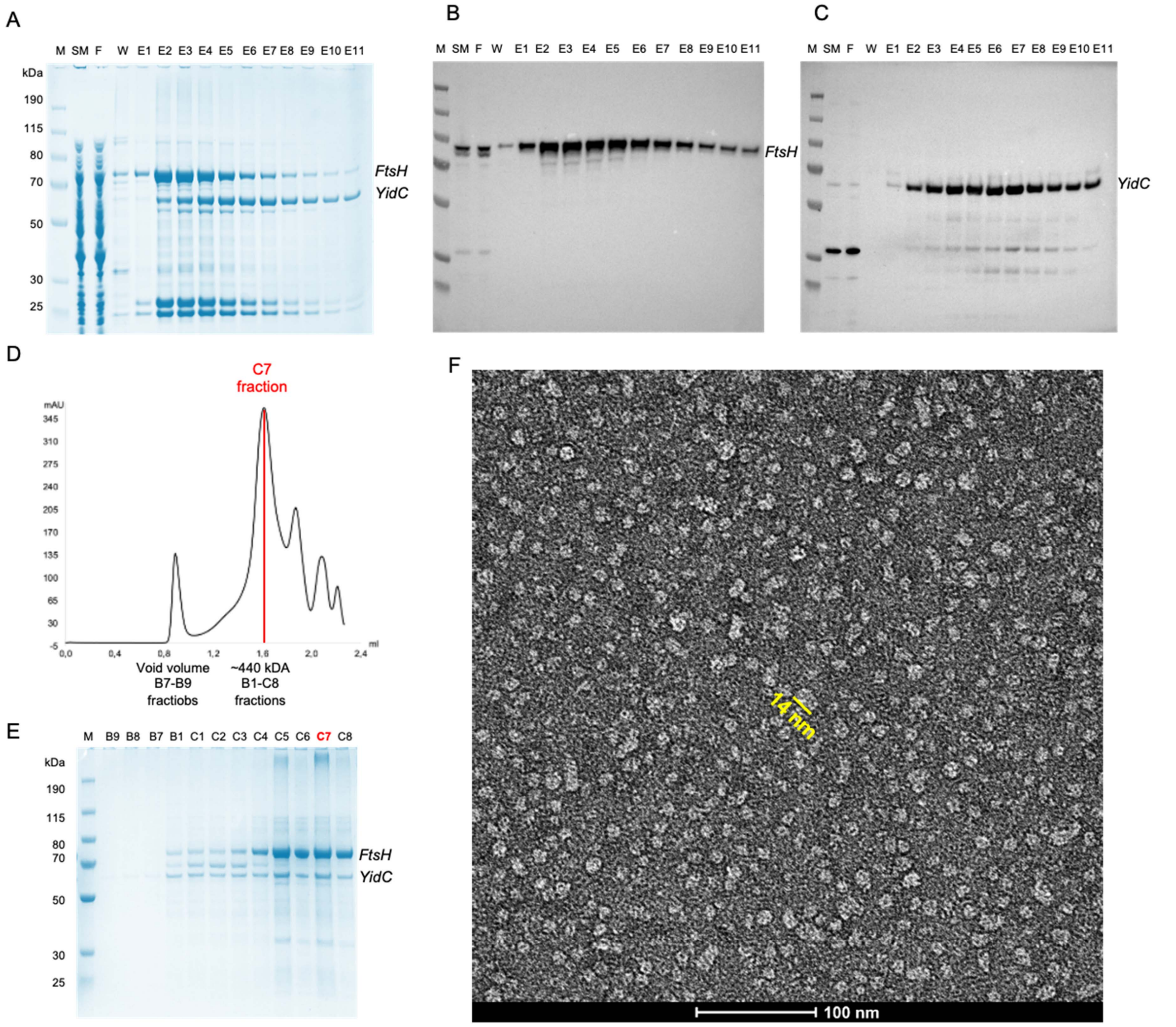

Figure S1 Purification of the FtsH–YidC sample with only DDM. (A) Coomassie stained SDS gel of Ni-NTA purified samples. (B) Western blot using anti-StrepTag II antibody to detect the presence of FtsH. (C) Western blot using anti-His antibody to detect His-tagged YidC. (D) Superose 6 Increase 3.2/300 SEC chromatogram from ÄKTAmicro. Fraction C7 was further analysed. (E) Coomassie-stained SDS gel of SEC fractions corresponding to first and second peak. (F) Negative-stain EM micrograph of C7 fraction. Scale bar of a particle: 14 nm.

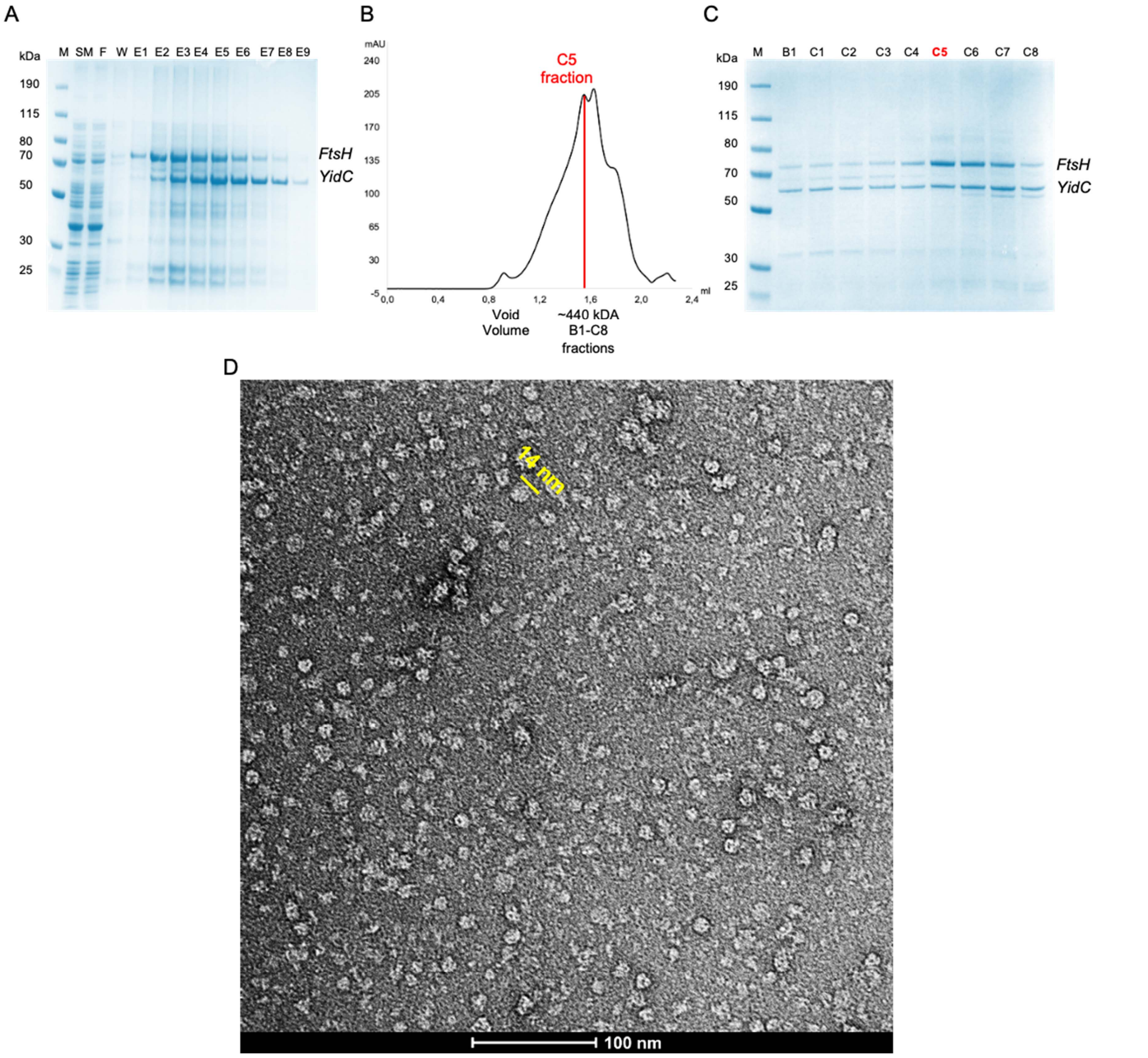

Figure S2 Purification of crosslinked FtsH–YidC sample with DDM-CHAPS combination. (A) Coomassie-stained SDS gel of Ni-NTA purified samples. (B) Superose 6 Increase 3.2/300 SEC chromatogram from ÄKTAmicro. Fraction C5 was further analysed. (C) Coomassie-stained SDS gel of SEC fractions. (D) Negative-stain EM micrograph of C5 fraction. Scale bar of a particle: 14 nm.

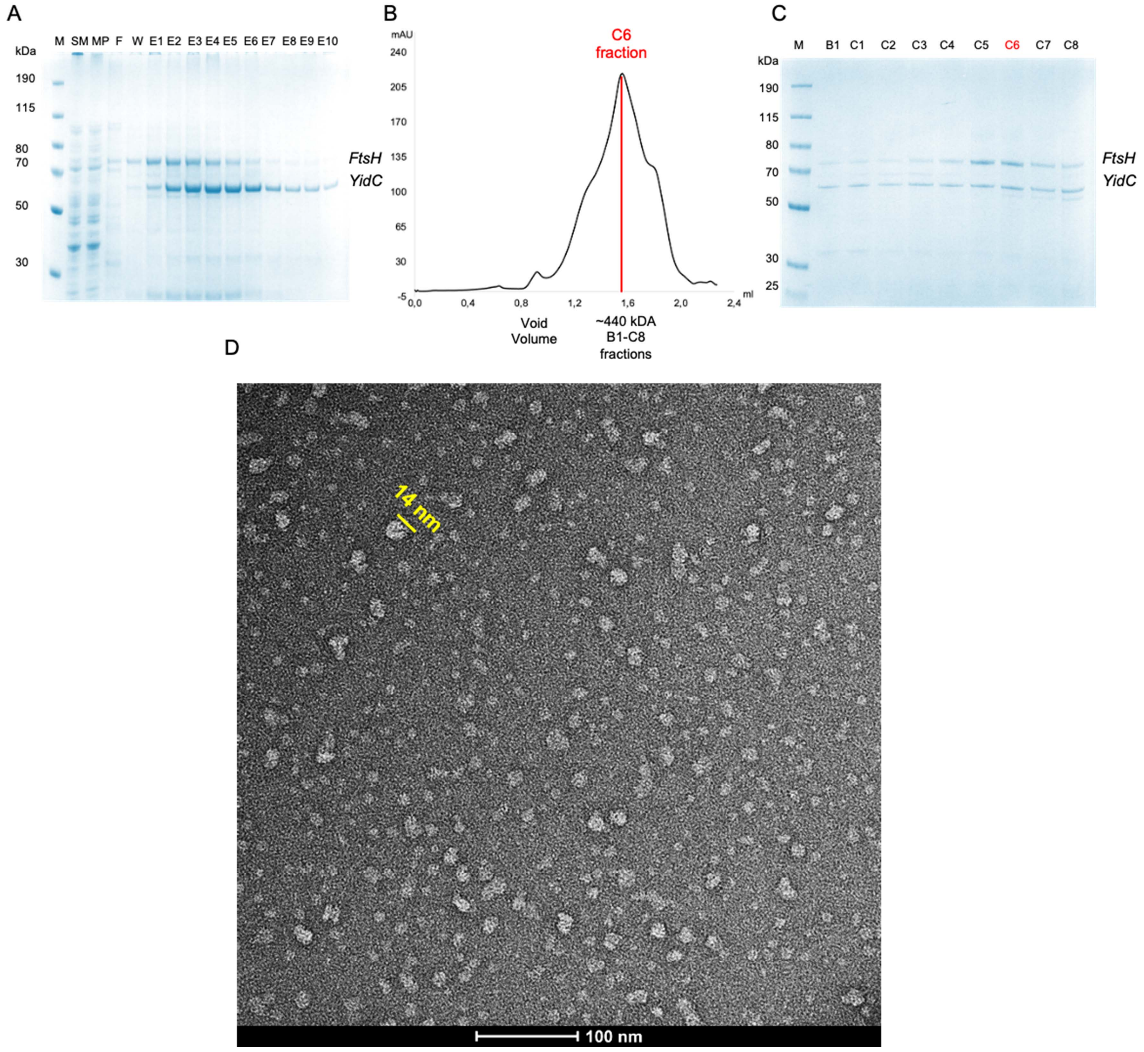

Figure S3 Purification of crosslinked FtsH–YidC sample with DDM-CHS combination**.** (A) Coomassie-stained SDS gel of Ni-NTA purified samples. (B) Superose 6 Increase 3.2/300 SEC chromatogram. Fraction C6 was further analysed. (C) Coomassie-stained SDS gel of SEC fractions. (D) Negative-stain EM micrograph of C5 fraction. Scale bar of a particle: 14 nm.

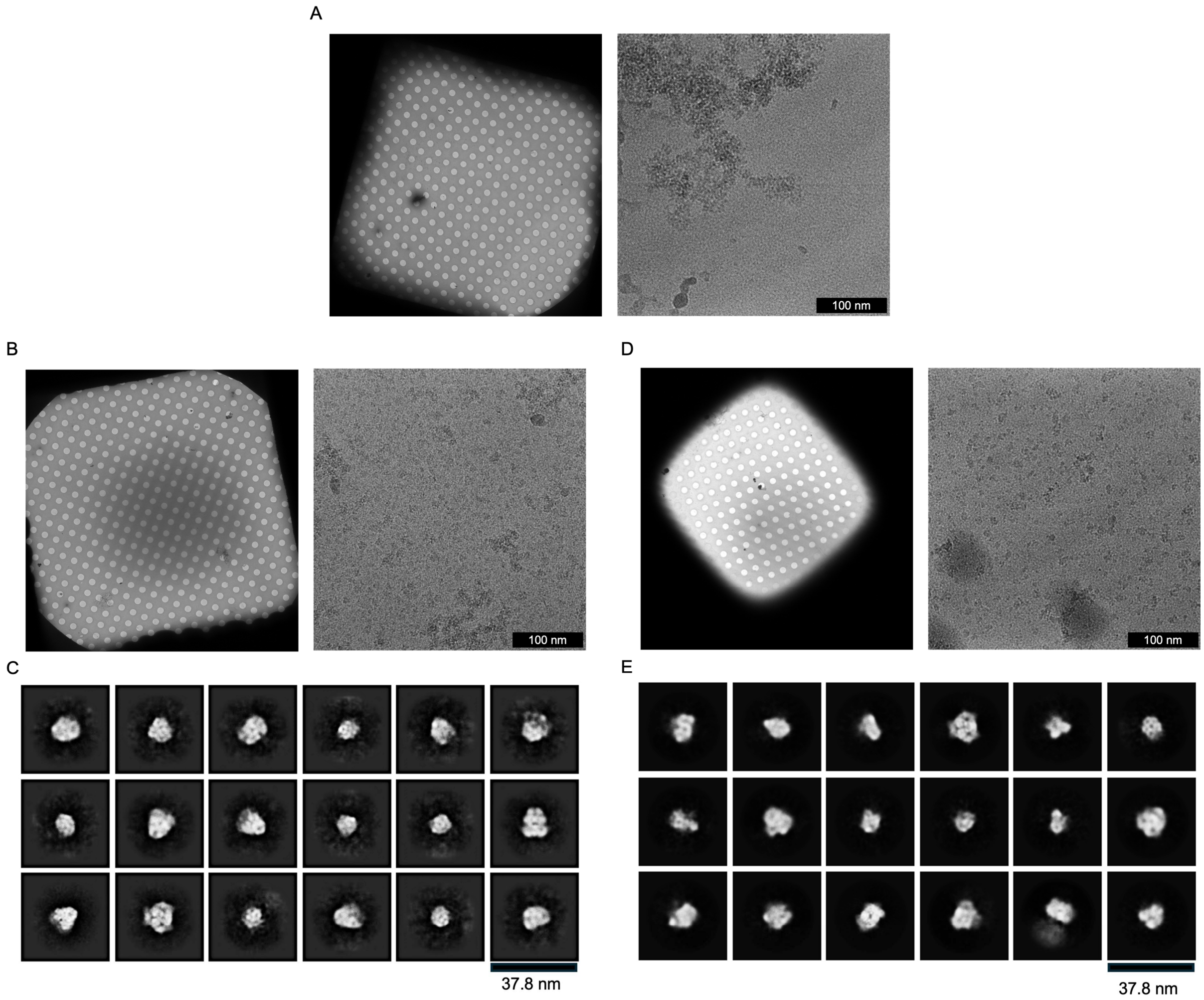

Figure S4 Cryo-EM screening of purified FtsH–YidC samples. (A) Micrograph of sample using C7 fraction after SEC with only DDM. (B) Micrograph of sample using C5 fraction after SEC with 0.4% w/v CHAPS as an additive prior to vitrification. Screening revealed dispersed particles suitable for further image analysis. (C) 2D class averages of C5 fraction with 0.4% w/v CHAPS processed by cryoSPARC. (D) Optimized micrograph after applying DDM-CHS combination for solubilization and purification. C6 fraction from SEC was used. Screening provided high-quality micrographs with well-dispersed particles suitable for data acquisition and downstream analysis. (E) 2D class averages of C6 fraction with DDM-CHS processed by cryoSPARC. Scale bar: 14 nm.

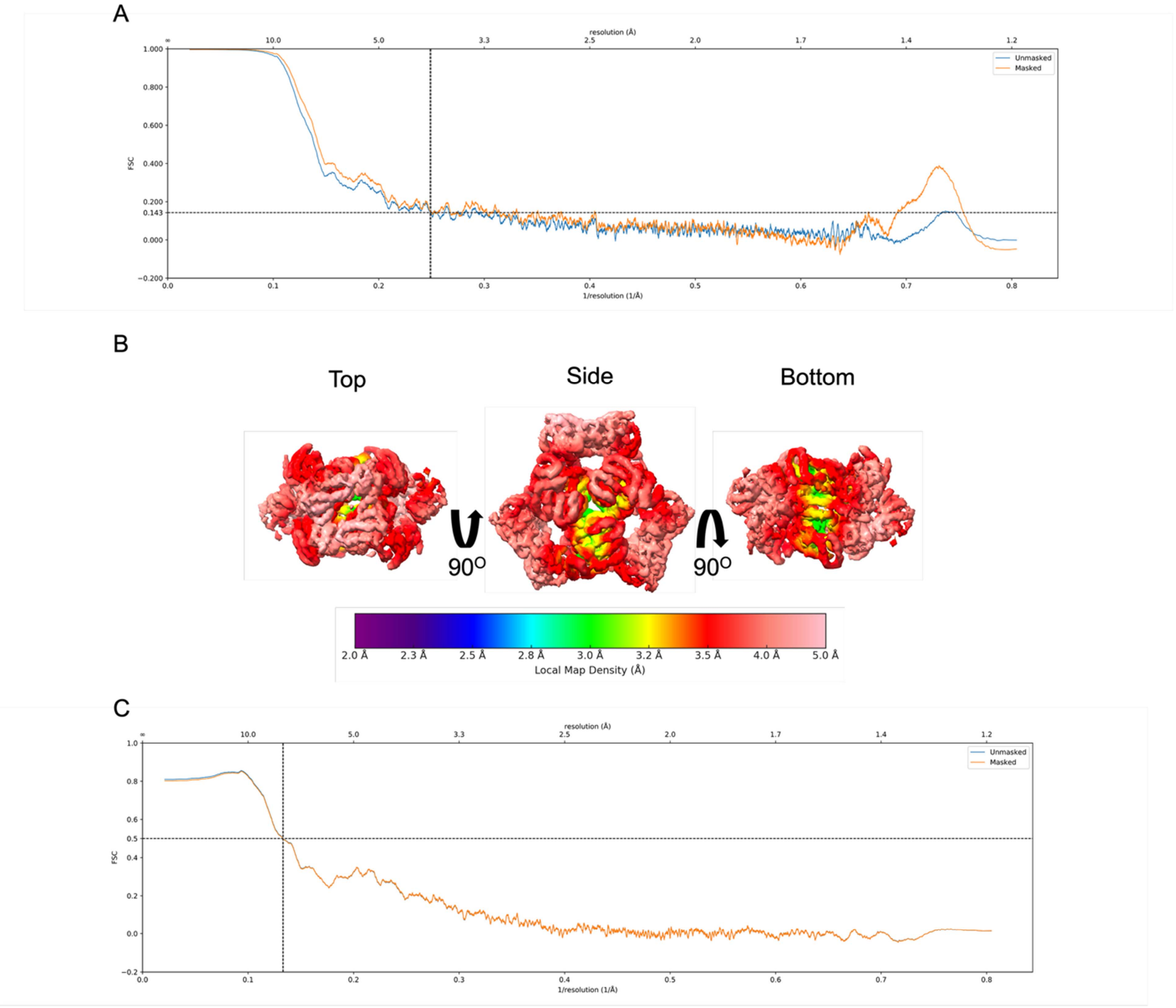

Figure S5 Quality of the ArnA map and model. (A) The Fourier Shell Correlation (FSC) curve for ArnA after refinement of ArnA particles. The FSC 0.143 criterion indicates an overall resolution of 4.0 Å. Blue curve: FSC curve of unmasked maps; orange curve: FSC curve of phase randomized masked maps. (B) Local resolution of the final ArnA cryo-EM map calculated in PHENIX. The core of the complex is resolved at 3.5 Å whereas peripheral parts have a lower resolution of ~ 4 Å. (C) FSC curve calculated between the ArnA atomic model and the final cryo-EM map. The map/model FSC at 0.5 reaches a resolution of 7.5 Å.

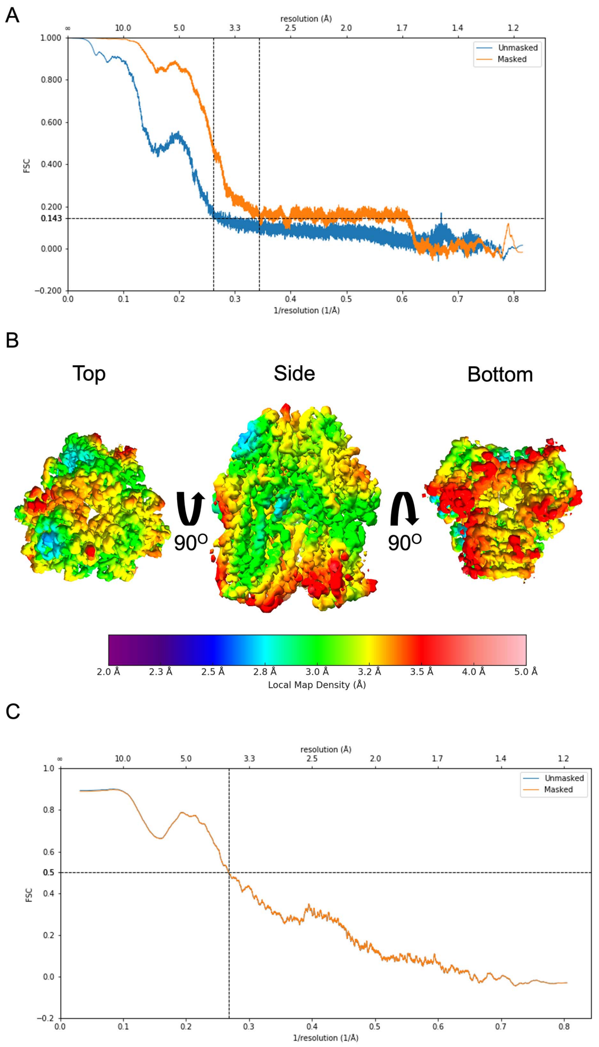

Figure S6 Quality of the AcrB map and model. (A) The Fourier Shell Correlation (FSC) curve for AcrB after gold-standard refinement of AcrB particles (blue curve). The FSC = 0.143 criterion indicates an overall resolution of 2.92 Å. Blue curve: FSC curve of unmasked maps; orange curve: FSC curve of phase randomized masked maps. (B) Local resolution of the final ArnA cryo-EM map calculated in PHENIX. The core of the complex is resolved at 3 Å whereas peripheral parts have a lower resolution of ~ 3.5 Å. (C) FSC curve calculated between the ArnA atomic model and the final cryo-EM map. The map/model FSC at 0.5 reaches a resolution of 3.7 Å.

A

B

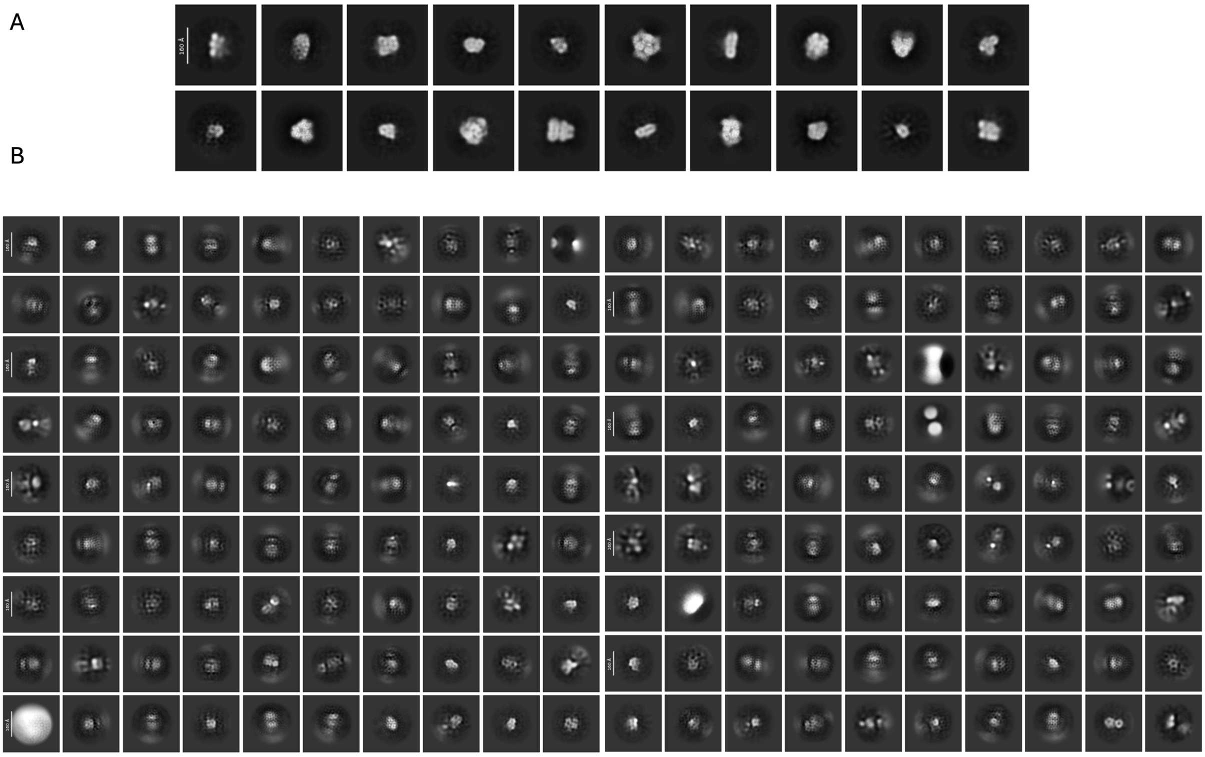

Figure S7 2D class averages of the Cryo-EM dataset after template picking. (A) Selected 2D class averages (1,262,383 particles) that were used for template-based particle picking. (B) Excluded 2D class averages (8,896,065 particles) that were discarded due to low resolution, anisotropy, or lack of defined structural features.

Table S1 Proteins co-purified with FtsH and YidC.

MS analysis of DSP-treated, SEC-purified FtsH-YidC sample purified from an *E. coli* C43(DE3) strain overexpressing FtsH, YidC, HflK and HflC (Caliseki et al., 2025). Target proteins are in red colour, off-target proteins detected in cryo-EM 2D class averages in green colour.

Accession Number	Protein Name
**P0AAI3**	**ATP-dependent zinc metalloprotease FtsH**
**P25714**	**Membrane protein insertase YidC**
**P0A6F5**	**Chaperonin GroEL**
P0A9Q7	Bifunctional alcohol/aldehyde dehydrogenase AdhE
P0A6Y8	Chaperone protein DnaK
P33599	NADH-quinone oxidoreductase subunit C
P0CE48	Elongation factor Tu (EF-Tu)
P60422	50S ribosomal protein L2
P0ABB4	ATP synthase subunit beta
P21513	Ribonuclease E (RNase E)
P0A910	Outer membrane protein A
P0AA10	50S ribosomal protein L13
P08622	Chaperone protein DnaJ
P0A6H5	ATP-dependent protease ATPase subunit hslU
P0AFG3	2-oxoglutarate dehydrogenase E1 component
P0AC41	Succinate dehydrogenase flavoprotein subunit
**P77398**	**Bifunctional polymyxin resistance protein ArnA**
P10408	Preprotein translocase subunit SecA
P0A7V8	30S ribosomal protein S4
P0A7V0	30S ribosomal protein S2
P36683	Aconitate hydratase B
P0ABC7	HflK protein
**P31224**	**Multidrug efflux pump subunit AcrB**
P0ACB7	Protein HemY
P0AFM6	Phage shock protein A
P0AG67	Small ribosomal subunit protein bS1
P09546	Bifunctional protein PutA
P0ABC3	HflC protein
P07014	Succinate dehydrogenase iron-sulfur subunit
